# State Cannabis Legalization and Psychosis-Related Health Care Utilization

**DOI:** 10.1001/jamanetworkopen.2022.52689

**Published:** 2023-01-25

**Authors:** Holly Elser, Keith Humphreys, Mathew V. Kiang, Swapnil Mehta, Jong H. Yoon, William O. Faustman, Ellicott C. Matthay

**Affiliations:** 1 Department of Neurology, Hospital of the University of Pennsylvania, Philadelphia; 2 Center for Population Health Sciences, Stanford University, Palo Alto, California; 3 Center for Innovation to Implementation, Veterans Affairs Palo Alto Health Care System, Palo Alto, California; 4 Department of Psychiatry and Behavioral Sciences, Stanford University School of Medicine, Palo Alto, California; 5 Epidemiology and Population Health, Stanford University School of Medicine, Palo Alto, California; 6 Division of Psychiatry, Veterans Affairs Palo Alto Health Care System, Palo Alto, California; 7 Department of Population Health, New York University Grossman School of Medicine, New York

## Abstract

**Importance:**

Psychosis is a hypothesized consequence of cannabis use. Legalization of cannabis could therefore be associated with an increase in rates of health care utilization for psychosis.

**Objective:**

To evaluate the association of state medical and recreational cannabis laws and commercialization with rates of psychosis-related health care utilization.

**Design, Setting, and Participants:**

Retrospective cohort design using state-level panel fixed effects to model within-state changes in monthly rates of psychosis-related health care claims as a function of state cannabis policy level, adjusting for time-varying state-level characteristics and state, year, and month fixed effects. Commercial and Medicare Advantage claims data for beneficiaries aged 16 years and older in all 50 US states and the District of Columbia, 2003 to 2017 were used. Data were analyzed from April 2021 to October 2022.

**Exposure:**

State cannabis legalization policies were measured for each state and month based on law type (medical or recreational) and degree of commercialization (presence or absence of retail outlets).

**Main Outcomes and Measures:**

Outcomes were rates of psychosis-related diagnoses and prescribed antipsychotics.

**Results:**

This study included 63 680 589 beneficiaries followed for 2 015 189 706 person-months. Women accounted for 51.8% of follow-up time with the majority of person-months recorded for those aged 65 years and older (77.3%) and among White beneficiaries (64.6%). Results from fully-adjusted models showed that, compared with no legalization policy, states with legalization policies experienced no statistically significant increase in rates of psychosis-related diagnoses (medical, no retail outlets: rate ratio [RR], 1.13; 95% CI, 0.97-1.36; medical, retail outlets: RR, 1.24; 95% CI, 0.96-1.61; recreational, no retail outlets: RR, 1.38; 95% CI, 0.93-2.04; recreational, retail outlets: RR, 1.39; 95% CI, 0.98-1.97) or prescribed antipsychotics (medical, no retail outlets RR, 1.00; 95% CI, 0.88-1.13; medical, retail outlets: RR, 1.01; 95% CI, 0.87-1.19; recreational, no retail outlets: RR, 1.13; 95% CI, 0.84-1.51; recreational, retail outlets: RR, 1.14; 95% CI, 0.89-1.45). In exploratory secondary analyses, rates of psychosis-related diagnoses increased significantly among men, people aged 55 to 64 years, and Asian beneficiaries in states with recreational policies compared with no policy.

**Conclusions and Relevance:**

In this retrospective cohort study of commercial and Medicare Advantage claims data, state medical and recreational cannabis policies were not associated with a statistically significant increase in rates of psychosis-related health outcomes. As states continue to introduce new cannabis policies, continued evaluation of psychosis as a potential consequence of state cannabis legalization may be informative.

## Key Points

**Question:**

Is state cannabis legalization or commercialization associated with increased rates of psychosis-related health care claims?

**Findings:**

In this cohort study of claims data from 63 680 589 beneficiaries from 2003 to 2017, there was no statistically significant difference in the rates of psychosis-related diagnoses or prescribed antipsychotics in states with medical or recreational cannabis policies compared with states with no such policy.

**Meaning:**

The findings of this study do not support an association between state policies legalizing cannabis and psychosis-related outcomes; further research into this topic may be informative.

## Teaser

This cohort study investigates whether state cannabis legalization is associated with psychosis-related healthcare utilization.

## Introduction

Psychosis has long been investigated as a potential consequence of cannabis use. Among Swedish conscripts followed from 1969 to 1983, Andréasson and colleagues^1,2^ found a 3-fold increased risk for schizophrenia associated with heavy cannabis use compared with nonusers. An association between cannabis use and psychosis has since been demonstrated in numerous longitudinal studies.^3,4,5,6,7,8,9,10,11,12,13,14,15,16^ Findings from experimental research, genome-wide association studies, and mendelian randomization studies further support a causal link between cannabis use and schizophrenia.^3,17,18,19^ Whether cannabis plays a causal role in the onset of psychosis nevertheless remains a point of controversy.^17,20^

In the US, an estimated 48.2 million people aged 12 years and older used cannabis at least once in 2019.^21^ As of June 2022, medical cannabis is legal in 38 states, and 19 permit recreational use.^22^ With legalization, the price of cannabis has decreased substantially.^23,24^ Simultaneously, the average THC content of herbal cannabis in the US increased markedly from 4% in 1996 to 17% in 2017.^25,26,27^ Past research on cannabis legalization in the US suggests a range of potential outcomes including decreased arrest rates,^28^ increased clearance rates for violent crimes,^29^ increased rates of cannabis use disorder,^30,31^ and increased rates of self-harm among men younger than 40 years.^32^ A limited number of studies have further identified increased rates of psychotic disorders associated with state and regional cannabis legalization in the US and with national policies in Canada and Portugal.^33,34,35,36^ As states continue to introduce cannabis legislation, a thorough and comprehensive understanding of their potential health outcomes is essential. Yet to our knowledge, no studies have examined trends in psychosis-related outcomes as a function of medical and recreational cannabis laws across all US states.

We evaluate the association of state cannabis legalization with rates of psychosis-related health care claims among privately insured individuals followed from 2003 to 2017. As the outcomes of cannabis policies may depend on the provisions included,^32,37^ we define a measure of state cannabis policy that considers both medical and recreational laws and identifies whether states permitted commercial sales through retail outlets. We hypothesized a priori that rates of psychosis-related diagnoses and prescribed antipsychotics would be increased in states with recreational policies and in those permitting commercial sales. As the health outcomes of state cannabis policies may differ within populations,^30,31,32^ we considered rates of psychosis-related claims by sex, age, and race and ethnicity.

## Methods

The Optum Clinformatics Data Mart Database is a deidentified commercial and Medicare Advantage claims database composed of more than 63 million unique individuals followed from January 1, 2003, to December 31, 2017. Study data included member enrollment data, diagnostic codes, and pharmacy claims deterministically linked across file types with a unique patient identifier. This study included all beneficiaries aged 16 years and older with at least 1 month of insurance eligibility during the study period.

In this retrospective cohort study, we leveraged a panel fixed-effects design—an extension of differences-in-differences—in which the state-month was the unit of analysis to evaluate the association of state cannabis policies with rates of psychosis-related health care claims.^38^ We counted the number of unique claims with psychosis-related diagnoses, prescribed antipsychotics, and enrolled individuals for each state-month of follow-up. These values were merged with time-varying categorical measures of state cannabis policy level and state-level demographic, social, and economic characteristics. This study was approved by the institutional review board at Stanford University and is reported per the Strengthening the Reporting of Observational Studies in Epidemiology (STROBE) reporting guidelines. The requirement for written informed consent was waived for this study by all participating institutions because data were deidentified. The analysis plan was prepared and preregistered in August 2021.^39^ Additions to the preregistered plan are summarized in eAppendix 1 in Supplement 1.

### State Cannabis Policy Level

Decriminalization removes criminal penalties for simple possession and use of cannabis. Past research suggests that decriminalization does not exert a sufficiently large effect on cannabis use rates to influence rates of psychotic disorders.^40^ This analysis therefore focuses on legalization, in which personal use; cultivation of cannabis; or its production, promotion, and sale is permitted.

As in prior research,^32^ we created a time-varying categorical variable reflecting the type of cannabis use permitted (medical or recreational) and whether retail outlets were open and operational. Cannabis legalization policies without retail outlets allowed only home-grown cannabis or had not yet implemented commercial sales.^41^ Data for recreational cannabis laws were derived from the Alcohol Policy Information System cannabis law database. Data for medical cannabis laws were derived from public research available through 2017.^42^ For each state-month, we assigned state cannabis policy levels as follows: no medical or recreational policy; medical only, no retail outlets; medical only, retail outlets; recreational, no retail outlets; or recreational, retail outlets. In all analyses, states with no medical or recreational policy (hereafter, “no policy”) served as the referent.

### Psychosis-Related Claims

Claims with psychosis-related diagnoses (hereafter, “diagnoses”) were identified using codes from the *International Classification of Diseases, Ninth* and *Tenth Revisions* (*ICD*-9 and *ICD*-10) and subclassified as: nonaffective psychoses; mood disorders with psychotic features; substance-related psychosis; and other psychosis (eTable 1 in Supplement 1). Prescribed antipsychotics were identified from outpatient pharmacy records and subclassified as first- or second-generation (eTable 2 in Supplement 1). Prescriptions were standardized such that a 30-day supply counted as 1 prescription.

### State-Level Characteristics

Time-varying state-level covariates included alcohol law stringency score^43^; the annual percentage of non-Hispanic Asian, non-Hispanic Black, non-Hispanic White, and Hispanic residents from the US Census (2002-2009) and American Community Survey (2010-2017); annual percent living in poverty and median income from the Small Area Income and Poverty Estimates Program; and monthly percent unemployed from the Bureau of Labor Statistics Local Area Unemployment Files. We also included the count of all unique claims and prescriptions for each state-month as a measure of overall utilization.

### Statistical Analyses

We used panel fixed-effects to model rates of psychosis-related health care claims as a function of state cannabis policy level.^38^ We used generalized negative binomial regression, as statistical testing of dispersion indicated it was more appropriate than Poisson or quasi-Poisson. Counts of psychosis-related diagnoses and prescribed antipsychotics were the outcomes of interest. The number of eligible beneficiaries for each state-month was specified as the offset to estimate rate ratios (RR).

All analyses included state fixed effects to account for potential confounding by time-invariant state characteristics. Calendar year fixed effects were included to address state-invariant secular trends including increased consumption of cannabis-containing products over time and potential discontinuities in psychosis-related claims introduced by the *ICD* transition. Month fixed effects were included to address potential seasonality in psychosis-related outcomes. We additionally adjusted for the previously-described time-varying state-level characteristics lagged by 1 year to ensure temporal order. For all analyses, we calculated 95% CIs with robust SEs to account for repeated observations within states over time. Statistical inferences are presented based on α = 0.05. Data were analyzed using R Statistical Software, version 4.0.0 (R Project for Statistical Computing), and analyses were completed from April 2021 to October 2022.

#### Secondary Analyses

To examine potential differences in psychosis-related outcomes across key population subgroups, we conducted analyses within strata defined by sex (men, women), age group (16-34; 35-54; 55-64; ≥65), categorical race and ethnicity, and within subgroups of diagnoses and prescriptions. Data on sex, age, race and ethnicity were all provided in beneficiary enrollment files, and details on the ascertainment of race data were not available for data retrieved from these files. Secondary analyses were exploratory, and therefore we did not adjust for multiple comparisons consistent with expert recommendations.^44^

#### Sensitivity Analyses

We conducted the following robustness checks: (1) we defined the sum of all unique claims and prescriptions for all health conditions as alternative offsets to account for changes in overall health care utilization; (2) we tested a 6-category exposure that separated recreational cannabis policies into those with and without THC dose-related restrictions^45,46^; (3) we tested a 3-category exposure variable (no policy, medical policy, recreational policy); (4) we restricted to state-months with some form of cannabis policy (ie, we excluded state-months that did not adopt any form of cannabis legalization over the study period, and set medical policies without retail outlets as the referent); (5) we conducted negative control analyses^47^ including use of the rate of all unique diagnoses and all unique prescriptions as negative control outcomes, hypothetical law changes at randomly assigned dates as a negative control exposure, and naloxone overdose prevention laws as a negative control exposure to assess potential residual confounding by factors that influence drug policy; (6) we implemented an alternative estimator proposed by Callaway and Sant’Anna that relaxes the typical assumption of panel fixed effects estimators that policy effects are constant over time and do not depend on the timing of legalization.^48^ (eAppendix 2 in Supplement 1).

## Results

**Figure 1.**
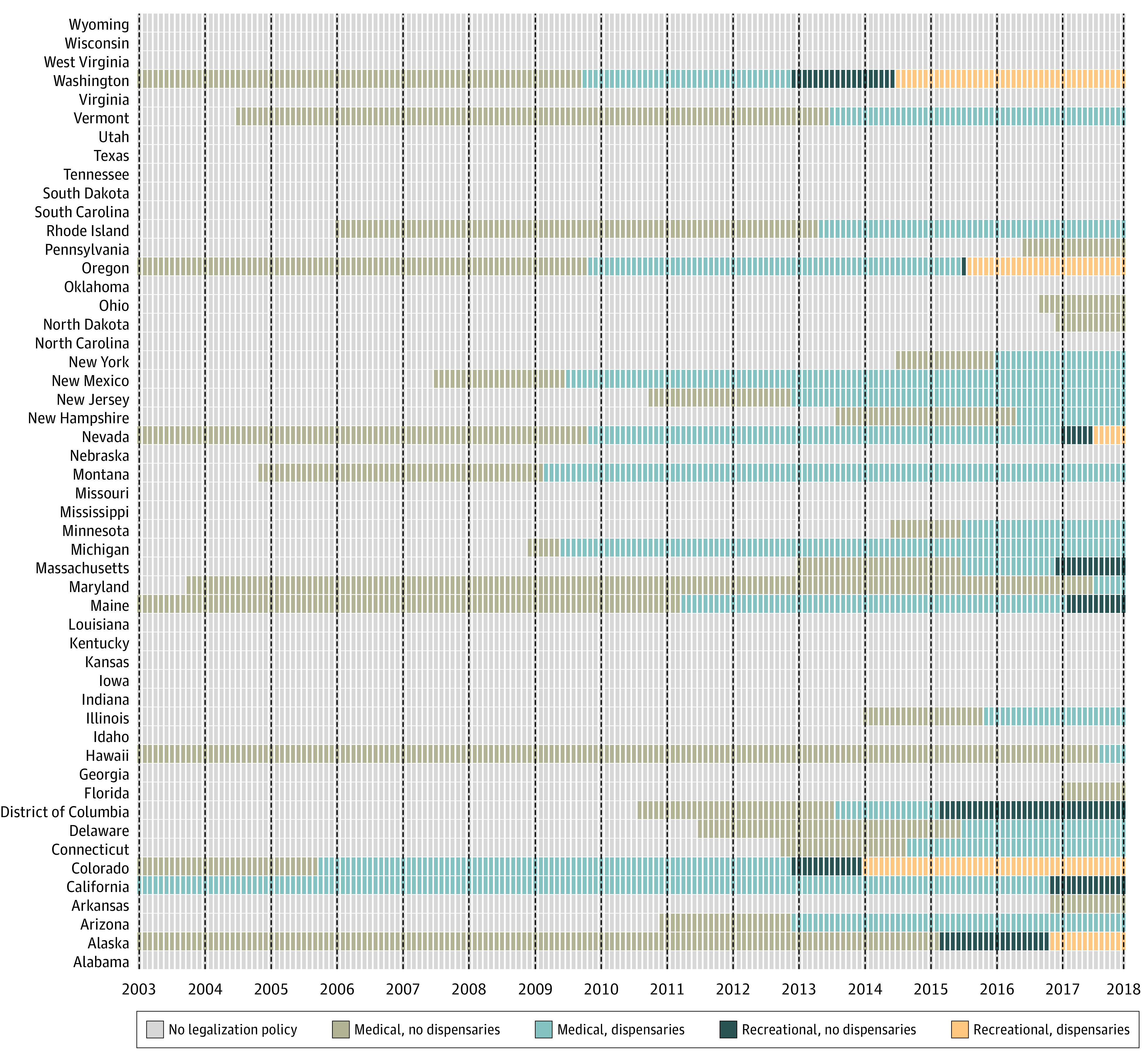
Classification of State Cannabis Policy Level by State, 2003-2017 Reproduced from “Evaluation of state cannabis laws and rates of self-harm and assault” (Matthay et al^32^).

This study included 63 680 589 beneficiaries with 2 015 189 706 total person-months of follow-up. Women accounted for 51.8% of follow-up time with 77.3% of person-months recorded among individuals 65 years or older and 64.6% among White beneficiaries. There were 7 503 907 psychosis-related diagnoses and 20 799 285 filled prescriptions for antipsychotics recorded over the study period. 29 states adopted medical or recreational cannabis legalization policies (Figure 1). Characteristics of the study population are presented in the Table. Additional state characteristics are summarized by state cannabis policy level in eTable 3 in Supplement 1.

**Table.** Demographic Characteristics, Overall and by Cannabis Policy Category

Characteristic	Person months, No. (%)					
	Overall (n = 2 015 189 706 )	No policy (n = 1 399 958 524)	Medical		Recreational	
			No dispensaries (n = 172 068 754)	Dispensaries (n = 370 695 920)	No dispensaries (n = 33 285 610)	Dispensaries (n = 39 180 898)
Sex^a^						
Male	971 559 233 (48.2)	677 785 592 (48.4)	82493727 (47.9)	176523088 (47.6)	15929852 (47.9)	18 826 974 (48.1)
Female	1 043 369 033 (51.8)	722 034 469 (51.6)	89476572 (52.0)	194152594 (52.4)	17353344 (52.1)	20 352 054 (51.9)
Age, y						
16-34	562 066 415 (27.9)	407 088 387 (29.1)	45 939 044 (26.7)	92 461 530 (24.9)	7 634 651 (22.9)	8942803 (22.8)
35-54	706 946 119 (35.1)	513 829 728 (36.7)	58 630 415 (34.1)	115 394 727 (31.1)	8 715 647 (26.2)	10 375 602 (26.5)
55-64	288 925 376 (14.3)	207 888 251 (14.8)	24 865 564 (14.5)	47 130 674 (12.7)	4 004 080 (12.0)	5 036 807 (12.9)
≥65	457 251 796 (22.7)	271 152 157 (19.4)	42 633 731 (24.8)	115 708 989 (31.2)	12 931 233 (33.8)	14 825 686 (37.9)
Race and ethnicity^b^						
Asian	85 493 072 (4.2)	41 409 520 (3.0)	8 971 711 (5.2)	30 394 285 (8.2)	2 935 649 (8.8)	1 781 906 (4.5)
Black	179 175 433 (8.9)	146 758 807 (10.5)	16 495 701 (9.6)	13 622 094 (3.7)	1 356 582 (4.1)	942 249 (2.4)
Hispanic	214 962 576 (10.7)	127 616 551 (9.1)	15 420 561 (9.0)	63 335 441 (17.1)	5 060 718 (15.2)	3 529 305 (9.0)
White	1 301 673 039 (64.6)	925 038 782 (66.1)	112 700 157 (65.5)	217 992 205 (58.8)	18 656 900 (56.1)	27 284 996 (69.6)
Diagnoses^c^	6 054 892 (100.0)	4 034 704 (66.6)	726 197 (12.0)	974 921 (16.1)	111 280 (1.8)	207 790 (3.4)
Prescriptions^d^	20 406 581 (100.0)	13 332 316 (65.3)	1 958 949 (9.6)	3 942 447 (19.3)	443 613 (2.2)	729 256 (3.6)

^a^ Sex was unknown for 261 440 person-months of follow-up.

^b^ Race and ethnicity were unknown for 233 885 586 person-months of follow-up.

^c^ Depicts the total number of psychosis-related diagnoses overall and by cannabis policy category.

^d^ Depicts the total number of filled prescriptions for antipsychotics overall and by cannabis policy category.

**Figure 2.**
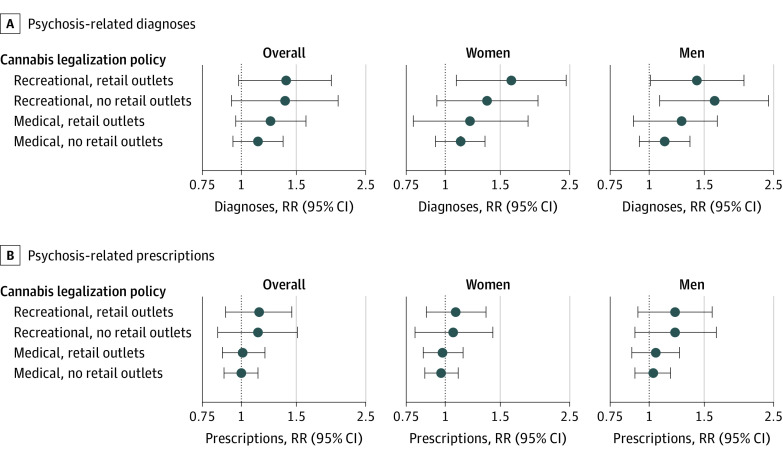
Adjusted Results for Rates of Psychosis-Related Diagnoses and Prescriptions by State Cannabis Policy Level, 2003 to 2017 Rate ratios were calculated using negative binomial models with person-months at risk as the offset. Models were adjusted for state-level confounders including percent non-Hispanic Black, non-Hispanic Asian, and Hispanic; percent unemployed and percent renting their home; median income; and the overall claims rate. We included fixed effects for state, year, and calendar month to address spatial and temporal autocorrelation. 95% CIs were calculated with robust SEs to account for repeated observations within states over time.

Crude rates were highest for state-months with recreational policies allowing retail outlets for both psychosis-related diagnoses (5.47 diagnoses per 1000 person-months of follow-up; 95% CI, 5.45-5.49) and prescribed antipsychotics (19.22 prescriptions per 1000 person-months of follow-up; 95% CI, 19.18-19.27) (eTable 4 in Supplement 1). Results from multivariate analysis showed no statistically significant increase in rates of psychosis-related diagnoses (medical, no retail outlets: RR, 1.13; 95% CI, 0.97-1.35; medical, with retail outlets: RR, 1.24; 95% CI, 0.96-1.61; recreational, no retail outlets: RR, 1.38; 95% CI, 0.93-2.04; recreational, with retail outlets: RR, 1.39; 95% CI, 0.98-1.97) or prescribed antipsychotics (medical, no retail outlets: RR, 1.00; 95% CI, 0.88-1.13; medical with retail outlets: RR, 1.01; 95% CI, 0.87-1.19; recreational, no retail outlets: RR, 1.13; 95% CI, 0.84-1.51; recreational with retail outlets: RR, 1.14; 95% CI, 0.89-1.45) vs states with no policy (Figure 2; eTable 5 in Supplement 1).

### Secondary Analyses

**Figure 3.**
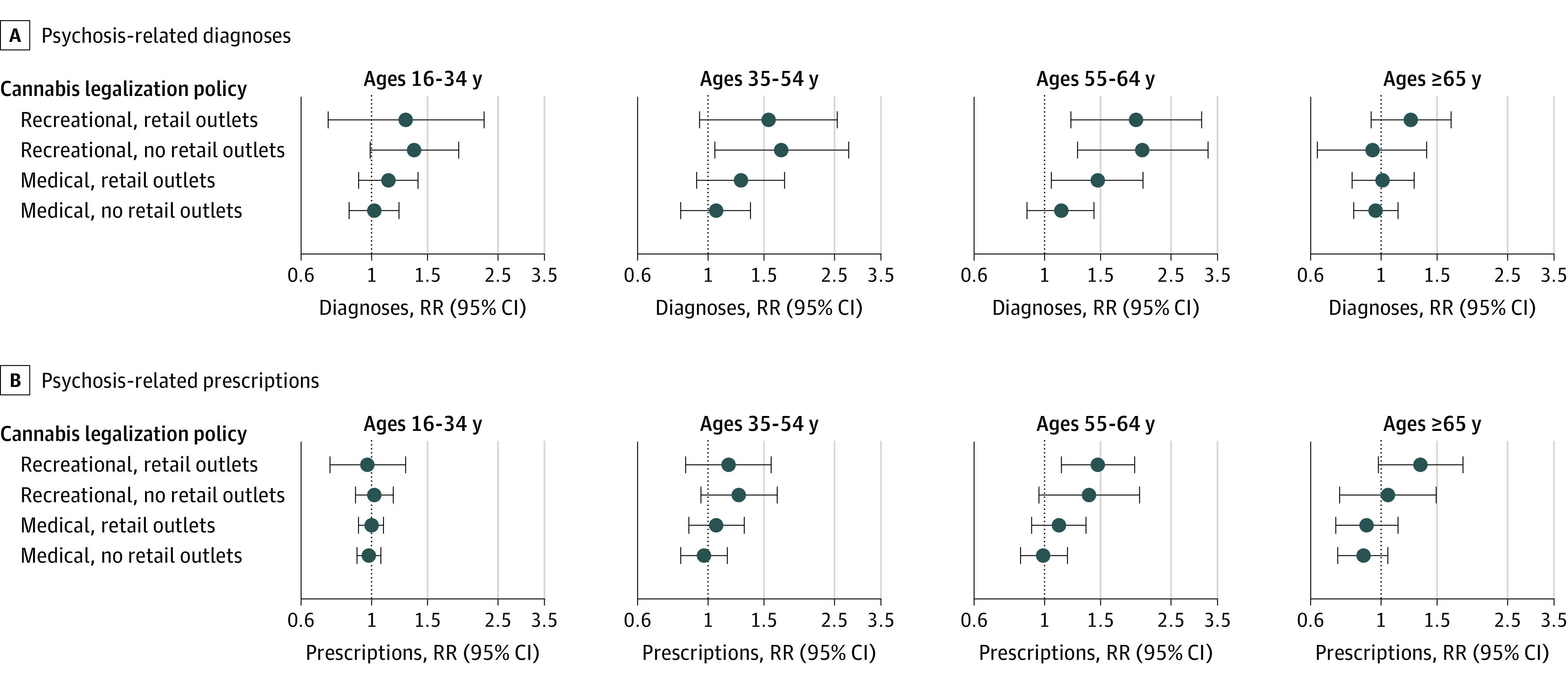
Adjusted Results for Rates of Psychosis-Related Diagnoses and Prescriptions by State Cannabis Policy Level by Age Group, 2003 to 2017 Rate ratios were calculated using negative binomial models with person-months at risk as the offset. Models were adjusted for state-level confounders including percent non-Hispanic Black, non-Hispanic Asian, and Hispanic; percent unemployed and percent renting their home; median income; and the overall claims rate. We included fixed effects for state, year, and calendar month to address spatial and temporal autocorrelation. 95% CIs were calculated with robust SEs to account for repeated observations within states over time.

**Figure 4.**
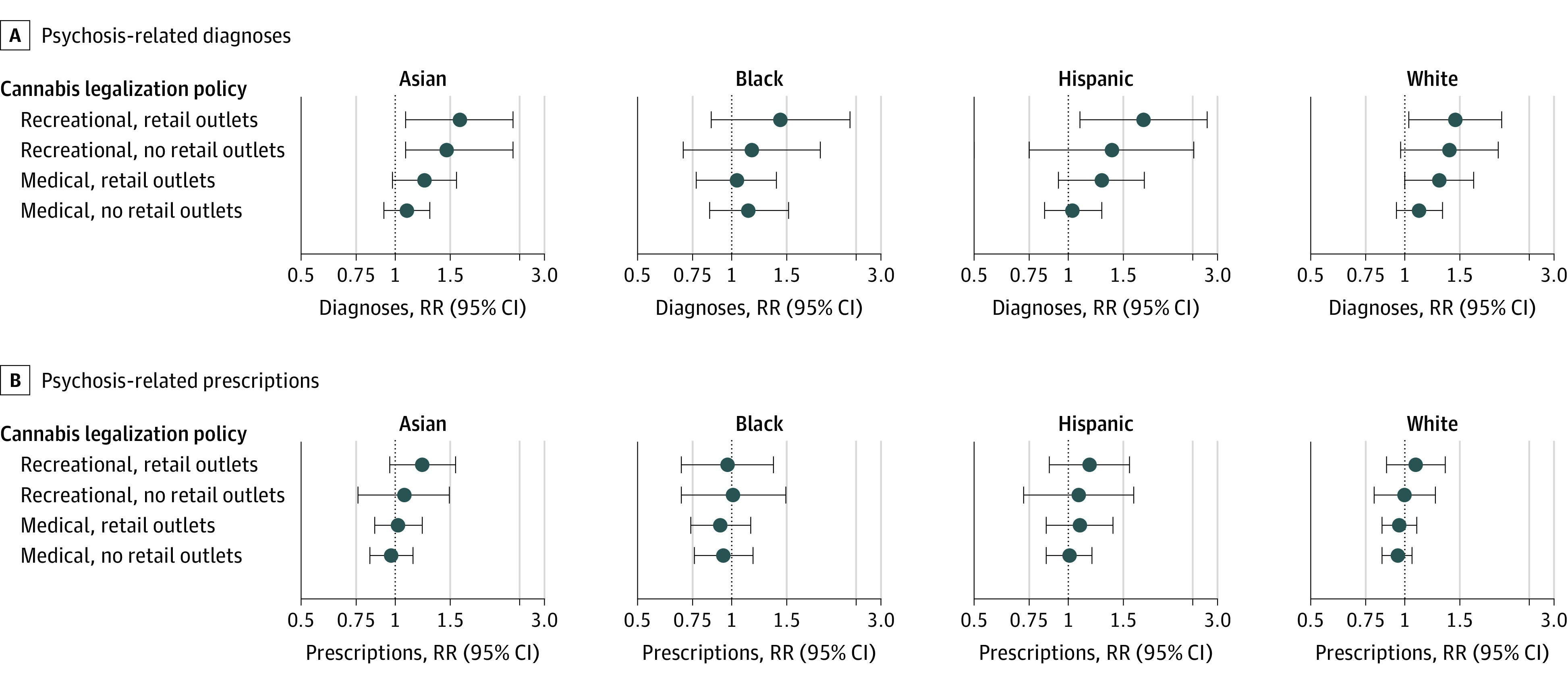
Adjusted Results for Rates of Psychosis-Related Diagnoses and Prescriptions at Varying Levels of Cannabis Commercialization by Race and Ethnicity, 2003 to 2017 Rate ratios were calculated using negative binomial models with person-months at risk as the offset. Models were adjusted for state-level confounders including percent non-Hispanic Black, non-Hispanic Asian, and Hispanic; percent unemployed and percent renting their home; median income; and the overall claims rate. We included fixed effects for state, year, and calendar month to address spatial and temporal autocorrelation. 95% CIs were calculated with robust standard errors to account for repeated observations within states over time. Because of sparsity of observations across covariate strata, for subgroup analysis among Asian beneficiaries we excluded observations from the 4 states with the fewest Asian beneficiaries (Vermont, South Dakota, Montana, and Alaska) to calculate cluster robust standard errors (0.09% of follow-up time in this subgroup).

In exploratory secondary analyses, rates of psychosis-related diagnoses were increased in states with recreational policies as compared with no policy for men (medical, no retail outlets: RR, 1.12; 95% CI, 0.93-1.35; medical with retail outlets: RR, 1.27; 95% CI, 0.89-1.65; recreational, no retail outlets: RR, 1.62; 95% CI, 1.08-2.41; recreational with retail outlets: RR, 1.42; 95% CI, 1.01-2.01) among those aged 55 to 64 years (medical, no retail outlets: RR, 1.13; 95% CI, 0.88-1.43; medical with retail outlets: RR, 1.47; 95% CI, 1.05-2.04; recreational, no retail outlets: RR, 2.03; 95% CI, 1.27-3.27; recreational with retail outlets: RR, 1.94; 95% CI, 1.21-3.12) and among Asian beneficiaries (medical, no retail outlets: RR, 1.09; 95% CI, 0.92-1.29; medical with retail outlets: RR, 1.24; 95% CI, 0.98-2.38; recreational, no retail outlets: RR, 1.46; 95% CI, 1.08-2.39; recreational with retail outlets: RR, 1.61; 95% CI, 1.08-2.38). We observed no statistically significant association with prescribed antipsychotics (Figure 2, Figure 3, and Figure 4; eTables 6, 7, 8, and 9 in Supplement 1). Analysis by diagnostic subgroup and for first vs second generation antipsychotics were consistent with those of our primary analysis (eTables 10 and 11 in Supplement 1).

### Sensitivity Analyses

Results of sensitivity analyses were generally consistent with our main analyses with alternative offsets; 3- and 6-category exposure metrics; and when analysis was restricted to state-months with some form of cannabis policy in place (eTables 12, 13, 14, and 15 in Supplement 1). Negative control analyses with unique diagnoses specified as the outcome of interest showed an inverse association for recreational policies with no retail outlets. We observed a dose-response pattern consistent with our primary analysis when unique prescriptions were specified as the outcome of interest (eTable 16 in Supplement 1). Negative control analyses with hypothetical law changes at randomly assigned dates showed no evidence of an association, as expected (eTable 17 in Supplement 1). We observed a small positive association when naloxone laws were assigned as the exposure of interest for both diagnoses (RR, 1.18; 95% CI, 1.05-1.32) and prescriptions (RR, 1.15; 95% CI, 1.07-1.25) (eTable 18 in Supplement 1). Using the Callaway and Sant’Anna estimator, we observed a pattern of positive associations for increasingly permissive state cannabis policies, consistent with our primary analysis (eTable 19 in Supplement 1).

## Discussion

Psychotic disorders are known to cause considerable personal hardship and may impede an individual’s ability to complete their education, maintain employment, and otherwise function as expected in society.^49^ This study is the first and largest, to our knowledge, to quantify the association of medical and recreational cannabis policies with rates of psychosis-related health care claims across US states. We found that state medical and recreational cannabis policies were not associated with a statistically significant increase in rates of psychosis-related health outcomes. In exploratory secondary analyses, rates of psychosis-related diagnoses increased significantly among men, people aged 55 to 64 years, and Asian beneficiaries in states with recreational policies compared with no policy.

A limited number of prior studies have generally reported increased rates of psychosis-related health outcomes in association with state and local cannabis policies. In Colorado, Hall et al^33^ analyzed administrative records from statewide emergency department (ED) visits following legalization of recreational cannabis from 2012 to 2014. They found a 9-fold increase in the prevalence of schizophrenia and other psychotic disorders in cannabis-associated ED visits compared with visits unrelated to cannabis. Using cross-sectional data from the 2017 National Inpatient Sample, Moran et al^34^ found that in the Pacific census division—where most states had introduced recreational cannabis policies by 2017—odds of psychosis-related hospitalization were higher than elsewhere in the US. Callaghan et al^36^ found that ED presentations for cannabis-induced psychosis in Ontario and Alberta doubled between April 2015 to December 2019 following legalization via the Cannabis Act on October 17, 2018. Finally, Gonçalves-Pinho et al^35^ reported an increase in the percentage of patients with a psychotic disorder and increases in cannabis use prevalence from 0.87 to 10.6% in Portugal in the 15 years following decriminalization.

Our analysis naturally extends the existing literature by leveraging prospectively recorded health care claims for the entire US. Whereas prior studies have focused on the effects of medical or recreational cannabis policies alone,^30,31,33,34^ our analysis considers both medical and recreational policies and whether commercial sales were permitted.^41^ Cannabis commercialization is hypothesized to magnify cannabis use and associated outcomes through reduced prices, widespread marketing, and expanded availability of high-potency cannabis-containing products.^24,50^ Prior studies have also demonstrated that associations depend on whether the policy permitted commercial sales.^32,37^

In contrast with these prior studies, we did not observe a statistically significant association of state cannabis policy level with overall rates of psychosis-related diagnoses or prescribed antipsychotics. Importantly, our outcome measures were composed of psychosis-related diagnosis codes associated with health care delivery, and therefore do not capture episodes of psychosis among individuals who do not receive treatment. Because we cannot reliably distinguish new from existing psychotic disorders using administrative data, it is possible that state cannabis legalization has differential effects on incident vs prevalent psychosis that our results do not reflect. As states continue to introduce cannabis policies, the implications of state cannabis legalization for psychotic disorders warrants continued study, particularly in data settings where direct measures of disease onset and severity are available.

Finally, our analysis included exploratory subgroup analysis by sex, age, and race and ethnicity. Racial and ethnic disparities have been reported less frequently in the literature on cannabis and psychosis,^23^ but are an important area for future study given the potential for differences in social class, norms around mental illness, material resources, access to mental health care, and clinician bias to create and perpetuate disparities. In analysis by sex and age, results were accentuated among men and among those aged 55 to 64 years. Past research identifies heavy cannabis use in adolescence as a salient risk factor for onset of psychosis in young adulthood,^51^ but little research has examined middle-aged and older adults. However, age is a significant predictor of mental health care utilization, and middle-aged adults are generally more likely to receive services than those at the extremes of age.^52^ More broadly, findings by sex, age, and race and ethnicity underscore the importance of continued examination of heterogeneous effects of cannabis policies and the implications of these differences for health inequities.

### Limitations

As psychotic disorders are associated with lower socioeconomic position,^53^ generalizability of our study findings is limited by our focus on insured individuals likely with fluctuating representativeness within states over the study period. We aimed to minimize confounding by controlling for state and time fixed effects and time-varying state-level characteristics, but residual confounding by factors associated with the broader policy environment such as expanded social safety net programs, rates of comorbid substance use, and preferential relocation by individuals predisposed to psychosis is possible. This is evidenced by the nonnull association of naloxone access laws (designated as a negative control exposure) with psychosis-related diagnoses and prescribed antipsychotics. In addition, a potential limitation of the study was the inability to assess how participant race was determined and the source of the categorizations.

The study period spans the period before and after the Affordable Care Act (ACA) was passed into law in 2010. We anticipate that potential confounding by passage of the ACA in our analysis is minimized. First, there is no clear evidence of an immediate effect of the ACA on state cannabis policy level as only 2 states (Delaware and New Jersey) and the District of Columbia introduced new state cannabis policy shortly after passage of the ACA. Second, our analysis includes fixed effects for calendar year. This means that any changes that applied to all states at the same time are controlled by design with these fixed effects. If there is residual confounding due to the ACA, it would be because the influence of the ACA differs in a time-varying and state-specific way

We did not adjust for multiple comparisons in exploratory secondary analyses. Although Bonferroni correction may have desirable properties when the sample size is large and a moderate number of tests are performed, we note the effective sample size in our analysis is much smaller than the number of claims because the unit of analysis is the state-month. Nevertheless, we acknowledge the potential risk of type I error (ie, false-positive results) without correction for multiple comparisons.^54,55^ Additionally, several unexpected secondary findings are not easily explained and warrant further consideration. These include the minimal association with recreational cannabis policies that permit retail outlets but make no THC dose-related restrictions, and the dose-response association between cannabis policy level and overall rates of prescriptions. Future analyses should explore systematic differences in state-level factors including prescribing patterns that may be correlated with cannabis policies.

## Conclusions

In this retrospective cohort study of commercial and Medicare Advantage claims data, state medical and recreational cannabis policies were not associated with a statistically significant increase in rates of psychosis-related health outcomes. As US states continue to legalize the use, production, promotion, or sale of cannabis, continued examination of the implications of state cannabis policies for psychotic disorders may be informative, particularly with study designs that yield precise estimates in high-risk population subgroups.

## Notes

Supplement 1. **eAppendix 1. **Preregistration **eAppendix 2. **Callaway and Sant’Anna Estimator **eTable 1. **Diagnostic Codes From the International Classification of Diseases, Ninth and Tenth Revision Used to Identify Psychosis **eTable 2. **Typical and Atypical Antipsychotics **eTable 3.** Distribution of State Characteristics Overall and by Policy Type, 2003 to 2017 **eTable 4.** Crude Rates for Psychosis-Related Diagnoses and Prescriptions, 2003 to 2017 **eTable 5. **Adjusted Rate Ratios for the Association Between State Cannabis Policy Level and Psychosis-Related Diagnoses and Prescribed Antipsychotics, 2003 to 2017 **eTable 6.** Adjusted Rate Ratios for the Association Between State Cannabis Policy Level and Psychosis-Related Diagnoses and Prescribed Antipsychotics by Sex, 2003 to 2017 **eTable 7. **Adjusted Rate Ratios for the Association Between State Cannabis Policy Level and Psychosis-Related Diagnoses and Prescribed Antipsychotics by Age Group, 2003 to 2017 **eTable 8. **Adjusted Rate Ratios for the Association Between State Cannabis Policy Level and Psychosis-Related Diagnoses and Prescribed Antipsychotics by Age Group, 2003 to 2017 **eTable 9. **Adjusted Rate Ratios for the Association Between State Cannabis Policy Level and Psychosis-Related Diagnoses and Prescribed Antipsychotics by Race and Ethnicity, 2003 to 2017 **eTable 10.** Adjusted Rate Ratios for Psychosis-Related Diagnoses by Subtype, 2003 to 2017 **eTable 11. **Adjusted Rate Ratios for the Association Between State Cannabis Policy Level for First-Generation And Second-Generation Antipsychotics, 2003 to 2017 **eTable 12. **Sensitivity Analyses With Total Diagnoses and Prescriptions as Offsets **eTable 13.** Sensitivity Analysis With Simplified 3-Category Exposure **eTable 14. **Sensitivity Analysis With Recreational Policies With 6-Category Exposure **eTable 15.** Sensitivity Analysis Restricted to State-Months With Policy in Place **eTable 16. **Negative Outcome Controls—All Diagnoses and All Prescriptions **eTable 17. **Negative Exposure Control—Hypothetical Law Change **eTable 18.** Negative Exposure Control—Naloxone Overdose Prevention Laws **eTable 19.** Estimated Average Effect of Treatment on Treated (ATT) Comparing Sequential Levels of State Cannabis Policies Using Methods Proposed by Callaway and Sant’Anna, 2003 to 2017

Supplement 2.
Data Sharing Statement
