# Error in Figure 2

**DOI:** 10.1001/jamanetworkopen.2023.5547

**Published:** 2023-03-17

In the Original Investigation titled “State Cannabis Legalization and Psychosis-Related Health Care Utilization,”^1^ published January 25, 2023, there were errors in Figure 2. The rate ratios and 95% confidence intervals for the top middle panel, which depicts the rate ratios for psychosis-related claims as a function of state cannabis policy level for women, were depicted incorrectly. The rate ratios for medical with retail outlets should have appeared as 1.20 (95% CI, 0.92-1.56) instead of 1.20 (95% CI, 0.79-1.84); for recreational with no retail outlets, 1.20 (95% CI, 0.79-1.84) instead of 1.36 (95% CI, 0.94-1.98); and for recreational with retail outlets, 1.36 (95% CI, 0.94-1.98) instead of 1.63 (95% CI, 1.09-2.44). This article has been corrected.^1^

## References

[1] Elser H, Humphreys K, Kiang MV, . State cannabis legalization and psychosis-related health care utilization. JAMA Netw Open. 2023;6(1):e2252689. doi:10.1001/jamanetworkopen.2022.52689 36696111 PMC9925044

