# Supplementary material for: State Cannabis Legalization and Psychosis-Related Health Care Utilization
Source: JAMA Netw Open. 2023 Jan 25;6(1):e2252689. doi: 10.1001/jamanetworkopen.2022.52689 (PMC9925044; doi:10.1001/jamanetworkopen.2022.52689)
Supplement: Supplement 1. — eAppendix 1. Preregistration eAppendix 2. Callaway and Sant’Anna Estimator eTable 1. Diagnostic Codes From the International Classification of Diseases, Ninth and Tenth Revision Used to Identify Psychosis eTable 2. Typical and Atypical Antipsychotics eTable 3. Distribution of State Characteristics Overall and by Policy Type, 2003 to 2017 eTable 4. Crude Rates for Psychosis-Related Diagnoses and Prescriptions, 2003 to 2017 eTable 5. Adjusted Rate Ratios for the Association Between State Cannabis Policy Level and Psychosis-Related Diagnoses and Prescribed Antipsychotics, 2003 to 2017 eTable 6. Adjusted Rate Ratios for the Association Between State Cannabis Policy Level and Psychosis-Related Diagnoses and Prescribed Antipsychotics by Sex, 2003 to 2017 eTable 7. Adjusted Rate Ratios for the Association Between State Cannabis Policy Level and Psychosis-Related Diagnoses and Prescribed Antipsychotics by Age Group, 2003 to 2017 eTable 8. Adjusted Rate Ratios for the Association Between State Cannabis Policy Level and Psychosis-Related Diagnoses and Prescribed Antipsychotics by Age Group, 2003 to 2017 eTable 9. Adjusted Rate Ratios for the Association Between State Cannabis Policy Level and Psychosis-Related Diagnoses and Prescribed Antipsychotics by Race and Ethnicity, 2003 to 2017 eTable 10. Adjusted Rate Ratios for Psychosis-Related Diagnoses by Subtype, 2003 to 2017 eTable 11. Adjusted Rate Ratios for the Association Between State Cannabis Policy Level for First-Generation And Second-Generation Antipsychotics, 2003 to 2017 eTable 12. Sensitivity Analyses With Total Diagnoses and Prescriptions as Offsets eTable 13. Sensitivity Analysis With Simplified 3-Category Exposure eTable 14. Sensitivity Analysis With Recreational Policies With 6-Category Exposure eTable 15. Sensitivity Analysis Restricted to State-Months With Policy in Place eTable 16. Negative Outcome Controls—All Diagnoses and All Prescriptions eTable 17. Negative Exposure Control—Hypothetical Law Change eTable [file jamanetwopen-e2252689-s001.pdf]

Supplemental Online Content

Elser H, Humphreys K, Kiang MV, et al. State cannabis legalization and psychosis-related health care utilization. *JAMA Netw Open*. 2023;6(1):e2252689. doi:10.1001/jamanetworkopen.2022.52689

eAppendix 1. Preregistration
eAppendix 2. Callaway and Sant'Anna Estimator
eTable 1. Diagnostic Codes From the International Classification of Diseases, Ninth and Tenth Revision Used to Identify Psychosis
eTable 2. Typical and Atypical Antipsychotics
eTable 3. Distribution of State Characteristics Overall and by Policy Type, 2003 to 2017
eTable 4. Crude Rates for Psychosis-Related Diagnoses and Prescriptions, 2003 to 2017
eTable 5. Adjusted Rate Ratios for the Association Between State Cannabis Policy Level and Psychosis-Related Diagnoses and Prescribed Antipsychotics, 2003 to 2017
eTable 6. Adjusted Rate Ratios for the Association Between State Cannabis Policy Level and Psychosis-Related Diagnoses and Prescribed Antipsychotics by Sex, 2003 to 2017
eTable 7. Adjusted Rate Ratios for the Association Between State Cannabis Policy Level and Psychosis-Related Diagnoses and Prescribed Antipsychotics by Age Group, 2003 to 2017
eTable 8. Adjusted Rate Ratios for the Association Between State Cannabis Policy Level and Psychosis-Related Diagnoses and Prescribed Antipsychotics by Age Group, 2003 to 2017
eTable 9. Adjusted Rate Ratios for the Association Between State Cannabis Policy Level and Psychosis-Related Diagnoses and Prescribed Antipsychotics by Race and Ethnicity, 2003 to 2017
eTable 10. Adjusted Rate Ratios for Psychosis-Related Diagnoses by Subtype, 2003 to 2017
eTable 11. Adjusted Rate Ratios for the Association Between State Cannabis Policy Level for First-Generation and Second-Generation Antipsychotics, 2003 to 2017
eTable 12. Sensitivity Analyses With Total Diagnoses and Prescriptions as Offsets
eTable 13. Sensitivity Analysis With Simplified 3-Category Exposure
eTable 14. Sensitivity Analysis With Recreational Policies With 6-Category Exposure
eTable 15. Sensitivity Analysis Restricted to State-Months With Policy in Place
eTable 16. Negative Outcome Controls—All Diagnoses and All Prescriptions
eTable 17. Negative Exposure Control—Hypothetical Law Change
eTable 18. Negative Exposure Control—Naloxone Overdose Prevention Laws
eTable 19. Estimated Average Effect of Treatment on Treated (ATT) Comparing Sequential Levels of State Cannabis Policies Using Methods Proposed by Callaway and Sant'Anna, 2003 to 2017

© 2023 Elser H et al. *JAMA Network Open.*

This supplemental material has been provided by the authors to give readers additional information about their work.

© 2023 Elser H et al. *JAMA Network Open.*

eAppendix 1. Preregistration

Pre-registration from August 2021 is available through Open Science Framework (10.17605/OSF.IO/2PS64). This pre-registration details the data structure as well as all planned primary, sensitivity, and secondary analyses. Changes and additions to the analytic plan detailed in this pre-registration are detailed below:

1. Because of potential non-independence of rates of diagnoses and claims within states over time, we calculated cluster robust standard errors for all primary, secondary, and sensitivity analyses. In subgroup analysis among Asian beneficiaries, we were unable to calculate cluster robust standard errors due to data sparsity across covariate strata when all U.S. states were included. We therefore excluded the four states (Arkansas, Montana, South Dakota, Vermont) with the fewest Asian beneficiaries. Collectively, these states contributed only 0.09% of all person-time among Asian beneficiaries over the 15-year study period. Coefficients (ORs) remained stable when observations from these states were excluded for psychosis-related diagnoses and prescribed antipsychotics.
2. We incorporated an additional sensitivity analysis in which we implemented an alternative estimator proposed by Callaway and Sant'Anna to examine the association between state cannabis policy level and psychosis-related diagnoses and prescriptions. This estimator required contrast of sequential policy levels (no policy versus medical with dispensaries; medical without dispensaries versus medical with dispensaries; medical with dispensaries versus recreational without outlets; and recreational without outlets versus recreational with outlets) to estimate associations on the additive scale. Justification and further details regarding this estimator are included in Appendix B.
3. We additionally included alcohol policy score as a covariate to control for potential confounding by state-level policy environment in all analyses.

© 2023 Elser H et al. *JAMA Network Open.*

eAppendix 2. Callaway and Sant'Anna Estimator

Standard panel fixed effects analyses require the assumption that the causal effect of the treatment on the outcome is constant over time and does not depend on (a) when the unit was first treated or (b) duration of treatment. Given that late-legalizing states learned from early-legalizing states, and given that the impacts of cannabis policies may take years to develop, because factors such as social norms and commercial markets evolve over time, these assumptions are plausibly violated for cannabis policies. For this reason, we conducted a sensitivity analysis using the estimator proposed by Callaway and Sant'Anna that allows for dynamic treatment effects. (Callaway and Sant'Anna, 2021) We estimated a separate treatment effect for each unique combination of time-at-first-treatment and time-since-treatment. To estimate the overall treatment effect, the separate treatment effects were then aggregated, with weights proportional to the number of units contributing to the corresponding estimate. Estimation was implemented using the "did" package in R. We specified the doubly robust estimation method, the not-yet-treated units as the referent category, and bootstrapped standard errors.

The Callaway and Sant'Anna estimator is built for binary treatments, however our primary exposure was defined as an ordered categorical variable to capture key policy dimensions. We therefore estimated a series of contrasts for each sequential policy level (no policy versus medical with dispensaries; medical without dispensaries versus medical with dispensaries; medical with dispensaries versus recreational without outlets; and recreational without outlets versus recreational with outlets). For each contrast, we restricted the analysis to the state-months with only those two policy levels, and conducted estimation on that subset of the data, allowing for unbalanced panel data. As permitted by the estimator, we controlled for state-level covariates measured at the time step immediately prior to treatment (i.e., time-invariant). This contrasts with our primary analytic specification, which involved control for time-varying covariates. Additionally, because the Callaway and Sant'Anna uses linear models, the resulting estimates are additive-scale rate differences. This contrasts with our primary analytic specification, which involved negative binomial regression and estimated rate ratios. Comparisons between the two sets of estimates must take these differences into account.

References

Callaway B, Sant'Anna PH. Difference-in-differences with multiple time periods. *Journal of Econometrics.* 2021;225(2):200-230.

eTable 1. Diagnostic Codes From the International Classification of Diseases, Ninth and Tenth Revision Used to Identify Psychosis

Category	Revision	Code	Description
Non-affective psychoses	ICD-9	295.0	Simple type schizophrenia
	ICD-9	295.00	Simple type schizophrenia, unspecified
	ICD-9	295.01	Simple type schizophrenia, subchronic
	ICD-9	295.02	Simple type schizophrenia, chronic
	ICD-9	295.03	Simple type schizophrenia, subchronic with acute exacerbation
	ICD-9	295.04	Simple type schizophrenia, chronic with acute exacerbation
	ICD-9	295.05	Simple type schizophrenia, in remission
	ICD-9	295.1	Disorganized type schizophrenia
	ICD-9	295.10	Disorganized type schizophrenia, unspecified
	ICD-9	295.11	Disorganized type schizophrenia, subchronic
	ICD-9	295.12	Disorganized type schizophrenia, chronic
	ICD-9	295.13	Disorganized type schizophrenia, subchronic with acute exacerbation
	ICD-9	295.14	Disorganized type schizophrenia, chronic with acute exacerbation
	ICD-9	295.15	Disorganized type schizophrenia, in remission
	ICD-9	295.2	Catatonic type schizophrenia
	ICD-9	295.20	Catatonic type schizophrenia, unspecified
	ICD-9	295.21	Catatonic type schizophrenia, subchronic
	ICD-9	295.22	Catatonic type schizophrenia, chronic
	ICD-9	295.23	Catatonic type schizophrenia, subchronic with acute exacerbation
	ICD-9	295.24	Catatonic type schizophrenia, chronic with acute exacerbation
	ICD-9	295.25	Catatonic type schizophrenia, in remission
	ICD-9	295.3	Paranoid type schizophrenia
	ICD-9	295.30	Paranoid type schizophrenia, unspecified
	ICD-9	295.31	Paranoid type schizophrenia, subchronic
	ICD-9	295.32	Paranoid type schizophrenia, chronic
	ICD-9	295.33	Paranoid type schizophrenia, subchronic with acute exacerbation
	ICD-9	295.34	Paranoid type schizophrenia, chronic with acute exacerbation
	ICD-9	295.35	Paranoid type schizophrenia, in remission
	ICD-9	295.4	Schizophreniform disorder
	ICD-9	295.40	Schizophreniform disorder, unspecified
	ICD-9	295.41	Schizophreniform disorder, subchronic
	ICD-9	295.42	Schizophreniform disorder, chronic
	ICD-9	295.43	Schizophreniform disorder, subchronic with acute exacerbation
	ICD-9	295.44	Schizophreniform disorder, chronic with acute exacerbation
	ICD-9	295.45	Schizophreniform disorder, in remission
	ICD-9	295.5	Latent schizophrenia
	ICD-9	295.50	Latent schizophrenia, unspecified
	ICD-9	295.51	Latent schizophrenia, subchronic
	ICD-9	295.52	Latent schizophrenia, chronic
	ICD-9	295.53	Latent schizophrenia, subchronic with acute exacerbation

© 2023 Elser H et al. *JAMA Network Open.*

Category	Revision	Code	Description
Non-affective psychoses	ICD-9	295.54	Latent schizophrenia, chronic with acute exacerbation
	ICD-9	295.55	Latent schizophrenia, in remission
	ICD-9	295.6	Schizophrenic disorder, residual type
	ICD-9	295.60	Schizophrenic disorders, residual type, unspecified
	ICD-9	295.61	Schizophrenic disorders, residual type, subchronic
	ICD-9	295.62	Schizophrenic disorders, residual type, chronic
	ICD-9	295.63	Schizophrenic disorders, residual type, subchronic with acute exacerbation
	ICD-9	295.64	Schizophrenic disorders, residual type, chronic with acute exacerbation
	ICD-9	295.65	Schizophrenic disorders, residual type, in remission
	ICD-9	295.7	Schizoaffective disorder
	ICD-9	295.70	Schizoaffective disorder, unspecified
	ICD-9	295.71	Schizoaffective disorder, subchronic
	ICD-9	295.72	Schizoaffective disorder, chronic
	ICD-9	295.73	Schizoaffective disorder, subchronic with acute exacerbation
	ICD-9	295.74	Schizoaffective disorder, chronic with acute exacerbation
	ICD-9	295.75	Schizoaffective disorder, in remission
	ICD-9	295.8	Other specified types of schizophrenia
	ICD-9	295.80	Other specified types of schizophrenia, unspecified
	ICD-9	295.81	Other specified types of schizophrenia, subchronic
	ICD-9	295.82	Other specified types of schizophrenia, chronic
	ICD-9	295.83	Other specified types of schizophrenia, subchronic with acute exacerbation
	ICD-9	295.84	Other specified types of schizophrenia, chronic with acute exacerbation
	ICD-9	295.85	Other specified types of schizophrenia, in remission
	ICD-9	295.9	Unspecified schizophrenia
	ICD-9	295.90	Unspecified schizophrenia, unspecified
	ICD-9	295.91	Unspecified schizophrenia, subchronic
	ICD-9	295.92	Unspecified schizophrenia, chronic
	ICD-9	295.93	Unspecified schizophrenia, subchronic with acute exacerbation
	ICD-9	295.94	Unspecified schizophrenia, chronic with acute exacerbation
	ICD-9	295.95	Unspecified schizophrenia, in remission
	ICD-9	298.8	Other and unspecified reactive psychosis
	ICD-9	298.88	Other and unspecified reactive psychosis
	ICD-9	298.89	Other and unspecified reactive psychosis
	ICD-10	F20	Schizophrenia
	ICD-10	F20.0	Paranoid schizophrenia
	ICD-10	F20.1	Disorganized schizophrenia
	ICD-10	F20.2	Catatonic schizophrenia
	ICD-10	F20.3	Undifferentiated schizophrenia
	ICD-10	F20.5	Residual schizophrenia
	ICD-10	F20.8	Other schizophrenia
	ICD-10	F20.81	Schizophreniform disorder
**Category**	**Revision**	**Code**	**Description**

© 2023 Elser H et al. *JAMA Network Open.*

	ICD-10	F2089	Other schizophrenia
Non-affective psychoses	ICD-10	F20.9	Schizophrenia, unspecified
	ICD-10	F21	Schizotypal disorder
	ICD-10	F23	Brief psychotic disorder
	ICD-10	F25	Schizoaffective disorders
	ICD-10	F25.0	Schizoaffective disorders, bipolar type
	ICD-10	F25.1	Schizoaffective disorders, depressive type
	ICD-10	F25.8	Other schizoaffective disorders
	ICD-10	F25.9	Schizoaffective disorder, unspecified
	ICD-10	F28	Other psychotic disorder not due to a substance or known physiological condition
	ICD-10	F29	Unspecified psychosis not due to a substance or known physiological condition
	ICD-9	291.3	Alcohol-induced psychotic disorder with hallucinations
Substance-related Psychoses	ICD-9	291.5	Alcohol-induced psychotic disorder with delusions
	ICD-9	292.1	Drug-induced psychotic disorders
	ICD-9	292.11	Drug-induced psychotic disorder with delusions
	ICD-9	292.12	Drug-induced psychotic disorder with hallucinations
	ICD-10	F10.15	Alcohol abuse with alcohol-induced psychotic disorder
	ICD-10	F10.150	Alcohol abuse with alcohol-induced psychotic disorder, with delusions
	ICD-10	F10.151	Alcohol abuse with alcohol-induced psychotic disorder, with hallucinations
	ICD-10	F10.159	Alcohol abuse with alcohol-induced psychotic disorder, unspecified
	ICD-10	F10.25	Alcohol dependence with alcohol-induced psychotic disorder
	ICD-10	F10.250	Alcohol dependence with alcohol-induced psychotic disorder, with delusions
	ICD-10	F10.251	Alcohol dependence with alcohol-induced psychotic disorder, with hallucinations
	ICD-10	F10.259	Alcohol dependence with alcohol-induced psychotic disorder, unspecified
	ICD-10	F10.35	Alcohol use, unspecified with alcohol-induced psychotic disorder
	ICD-10	F10.350	Alcohol use, unspecified with alcohol-induced psychotic disorder, with delusions
	ICD-10	F10.351	Alcohol use, unspecified with alcohol-induced psychotic disorder, with hallucinations
	ICD-10	F10.359	Alcohol use, unspecified with alcohol-induced psychotic disorder, unspecified
	ICD-10	F11.15	Opioid abuse with opioid-induced psychotic disorder
	ICD-10	F11.150	Opioid abuse with opioid-induced psychotic disorder, with delusions
	ICD-10	F11.151	Opioid abuse with opioid-induced psychotic disorder, with hallucinations
	ICD-10	F11.159	Opioid abuse with opioid-induced psychotic disorder, unspecified
	ICD-10	F11.25	Opioid dependence with opioid-induced psychotic disorder
	ICD-10	F11.250	Opioid dependence with opioid-induced psychotic disorder, with delusions
	ICD-10	F11.251	Opioid dependence with opioid-induced psychotic disorder, with hallucinations
	ICD-10	F11.259	Opioid dependence with opioid-induced psychotic disorder, unspecified
	ICD-10	F11.95	Opioid use, unspecified with opioid-induced psychotic disorder
	ICD-10	F11.950	Opioid use, unspecified with opioid-induced psychotic disorder, with delusions
	ICD-10	F11.951	Opioid use, unspecified with opioid-induced psychotic disorder, with hallucinations
	ICD-10	F11.959	Opioid use, unspecified with opioid-induced psychotic disorder, unspecified
	ICD-10	F12.15	Cannabis abuse with psychotic disorder
**Category**	**Revision**	**Code**	**Description**
	ICD-10	F12.150	Cannabis abuse with psychotic disorder, with delusions

© 2023 Elser H et al. *JAMA Network Open.*

Substance-related Psychoses	ICD-10	F12.151	Cannabis abuse with psychotic disorder, with hallucinations
	ICD-10	F12.159	Cannabis abuse with psychotic disorder, unspecified
	ICD-10	F12.25	Cannabis dependence with psychotic disorder
	ICD-10	F12.250	Cannabis dependence with psychotic disorder, with delusions
	ICD-10	F12.251	Cannabis dependence with psychotic disorder, with hallucinations
	ICD-10	F12.259	Cannabis dependence with psychotic disorder, unspecified
	ICD-10	F12.95	Cannabis use, unspecified with psychotic disorder
	ICD-10	F12.950	Cannabis use, unspecified with psychotic disorder, with delusions
	ICD-10	F12.951	Cannabis use, unspecified with psychotic disorder, with hallucinations
	ICD-10	F12.952	Cannabis use, unspecified with psychotic disorder, unspecified
	ICD-10	F13.15	Sedative, hypnotic or anxiolytic abuse with sedative, hypnotic or anxiolytic-induced psychotic disorder
	ICD-10	F13.150	Sedative, hypnotic or anxiolytic abuse with sedative, hypnotic or anxiolytic-induced psychotic disorder, with delusions
	ICD-10	F13.151	Sedative, hypnotic or anxiolytic abuse with sedative, hypnotic or anxiolytic-induced psychotic disorder, with hallucinations
	ICD-10	F13.159	Sedative, hypnotic or anxiolytic abuse with sedative, hypnotic or anxiolytic-induced psychotic disorder, unspecified
	ICD-10	F13.25	Sedative, hypnotic or anxiolytic dependence with sedative, hypnotic or anxiolytic-induced psychotic disorder
	ICD-10	F13.250	Sedative, hypnotic or anxiolytic dependence with sedative, hypnotic or anxiolytic-induced psychotic disorder, with delusions
	ICD-10	F13.251	Sedative, hypnotic or anxiolytic dependence with sedative, hypnotic or anxiolytic-induced psychotic disorder, with hallucinations
	ICD-10	F13.259	Sedative, hypnotic or anxiolytic dependence with sedative, hypnotic or anxiolytic-induced psychotic disorder, unspecified
	ICD-10	F13.95	Sedative, hypnotic or anxiolytic use, unspecified with sedative, hypnotic or anxiolytic-induced psychotic disorder
	ICD-10	F13.950	Sedative, hypnotic or anxiolytic use, unspecified with sedative, hypnotic or anxiolytic-induced psychotic disorder, with delusions
	ICD-10	F13.951	Sedative, hypnotic or anxiolytic use, unspecified with sedative, hypnotic or anxiolytic-induced psychotic disorder, w/hallucinations
	ICD-10	F13.959	Sedative, hypnotic or anxiolytic use, unspecified with sedative, hypnotic or anxiolytic-induced psychotic disorder, unspecified
	ICD-10	F14.15	Cocaine abuse with cocaine-induced psychotic disorder
	ICD-10	F14.150	Cocaine abuse with cocaine-induced psychotic disorder, with delusions
	ICD-10	F14.151	Cocaine abuse with cocaine-induced psychotic disorder, with hallucinations
	ICD-10	F14.159	Cocaine abuse with cocaine-induced psychotic disorder, unspecified
	ICD-10	F14.25	Cocaine dependence with cocaine-induced psychotic disorder
	ICD-10	F14.250	Cocaine dependence with cocaine-induced psychotic disorder, with delusions
	ICD-10	F14.251	Cocaine dependence with cocaine-induced psychotic disorder, with hallucinations
	ICD-10	F14.259	Cocaine dependence with cocaine-induced psychotic disorder, unspecified
	ICD-10	F14.95	Cocaine use, unspecified with cocaine-induced psychotic disorder
	ICD-10	F14.950	Cocaine use, unspecified with cocaine-induced psychotic disorder, with delusions
	ICD-10	F14.951	Cocaine use, unspecified with cocaine-induced psychotic disorder, with hallucinations
	ICD-10	F14.959	Cocaine use, unspecified with cocaine-induced psychotic disorder, unspecified
	ICD-10	F15.15	Other stimulant abuse with stimulant-induced psychotic disorder
	ICD-10	F15.150	Other stimulant abuse with stimulant-induced psychotic disorder, with delusions
	ICD-10	F15.151	Other stimulant abuse with stimulant-induced psychotic disorder, with hallucinations
	ICD-10	F15.159	Other stimulant abuse with stimulant-induced psychotic disorder, unspecified
	ICD-10	F15.25	Other stimulant dependence with stimulant-induced psychotic
	ICD-10	F15.250	Other stimulant dependence with stimulant-induced psychotic, with delusions
**Category**	**Revision**	**Code**	**Description**
	ICD-10	F15.251	Other stimulant dependence with stimulant-induced psychotic, with hallucinations
	ICD-10	F15.259	Other stimulant dependence with stimulant-induced psychotic, unspecified

© 2023 Elser H et al. *JAMA Network Open.*

Substance-related psychosis	ICD-10	F15.95	Other stimulant use, unspecified with stimulant-induced psychotic disorder
	ICD-10	F15.950	Other stimulant use, unspecified with stimulant-induced psychotic disorder, with delusions
	ICD-10	F15.951	Other stimulant use, unspecified with stimulant-induced psychotic disorder, with hallucinations
	ICD-10	F15.959	Other stimulant use, unspecified with stimulant-induced psychotic disorder, unspecified
	ICD-10	F16.15	Hallucinogen abuse with hallucinogen-induced psychotic disorder
	ICD-10	F16.150	Hallucinogen abuse with hallucinogen-induced psychotic disorder, with delusions
	ICD-10	F16.151	Hallucinogen abuse with hallucinogen-induced psychotic disorder, with hallucinations
	ICD-10	F16.159	Hallucinogen abuse with hallucinogen-induced psychotic disorder, unspecified
	ICD-10	F16.25	Hallucinogen dependence with hallucinogen-induced psychotic disorder
	ICD-10	F16.250	Hallucinogen dependence with hallucinogen-induced psychotic disorder,with delusions
	ICD-10	F16.251	Hallucinogen dependence with hallucinogen-induced psychotic disorder, with hallucinations
	ICD-10	F16.259	Hallucinogen dependence with hallucinogen-induced psychotic disorder, unspecified
	ICD-10	F16.95	Hallucinogen use, unspecified with hallucinogen-induced psychotic disorder
	ICD-10	F16.950	Hallucinogen use, unspecified with hallucinogen-induced psychotic disorder, with delusions
	ICD-10	F16.951	Hallucinogen use, unspecified with hallucinogen-induced psychotic disorder, with hallucinations
	ICD-10	F16.959	Hallucinogen use, unspecified with hallucinogen-induced psychotic disorder, unspecified
	ICD-10	F18.15	Inhalant abuse with inhalant-induced psychotic disorder
	ICD-10	F18.150	Inhalant abuse with inhalant-induced psychotic disorder, with delusions
	ICD-10	F18.151	Inhalant abuse with inhalant-induced psychotic disorder, with hallucinations
	ICD-10	F18.159	Inhalant abuse with inhalant-induced psychotic disorder, unspecified
	ICD-10	F18.25	Inhalant dependence with inhalant-induced psychotic disorder
	ICD-10	F18.250	Inhalant dependence with inhalant-induced psychotic disorder, with delusions
	ICD-10	F18.251	Inhalant dependence with inhalant-induced psychotic disorder, with hallucinations
	ICD-10	F18.259	Inhalant dependence with inhalant-induced psychotic disorder, unspecified
	ICD-10	F18.95	Inhalant use, unspecified with inhalant-induced psychotic disorder
	ICD-10	F18.950	Inhalant use, unspecified with inhalant-induced psychotic disorder, with delusions
	ICD-10	F18.951	Inhalant use, unspecified with inhalant-induced psychotic disorder, with hallucinations
	ICD-10	F18.959	Inhalant use, unspecified with inhalant-induced psychotic disorder, unspecified
	ICD-10	F19.15	Other psychoactive substance abuse with psychoactive substance-induced psychotic disorder
	ICD-10	F19.150	Other psychoactive substance abuse with psychoactive substance-induced psychotic disorder, with delusions
	ICD-10	F19.151	Other psychoactive substance abuse with psychoactive substance-induced psychotic disorder, with hallucinations
	ICD-10	F19.159	Other psychoactive substance abuse with psychoactive substance-induced psychotic disorder, unspecified
	ICD-10	F19.25	Other psychoactive substance dependence with psychoactive substance-induced psychotic disorder
	ICD-10	F19.250	Other psychoactive substance dependence with psychoactive substance-induced psychotic disorder, with delusions
	ICD-10	F19.251	Other psychoactive substance dependence with psychoactive substance-induced psychotic disorder, with hallucinations
	ICD-10	F19.259	Other psychoactive substance dependence with psychoactive substance-induced psychotic disorder, unspecified
	ICD-10	F19.95	Other psychoactive substance use, unspecified with psychoactive substance-induced psychotic disorder
	ICD-10	F19.950	Other psychoactive substance use, unspecified with psychoactive substance-induced psychotic disorder, with delusions
	ICD-10	F19.951	Other psychoactive substance use, unspecified with psychoactive substance-induced psychotic disorder, with hallucinations
**Category**	**Revision**	**Code**	**Description**
Substance-related psychosis	ICD-10	F19.959	Other psychoactive substance use, unspecified with psychoactive substance-induced psychotic disorder, unspecified
	ICD-9	296.04	Bipolar I disorder, single manic episode, severe, specified as with psychotic behavior

© 2023 Elser H et al. *JAMA Network Open.*

Mood disorders with psychotic features	ICD-9	296.14	Manic affective disorder, recurrent episode, severe, specified as with psychotic behavior
	ICD-9	296.24	Major depressive affective disorder, single episode, severe, specified as with psychotic behavior
	ICD-9	296.34	Major depressive affective disorder, recurrent episode, severe, specified as with psychotic behavior
	ICD-9	296.44	Bipolar I disorder, most recent episode (or current) manic, severe, specified as with psychotic behavior
	ICD-9	296.54	Bipolar I disorder, most recent episode (or current) depressed, severe, specified as with psychotic behavior
	ICD-9	296.64	Bipolar I disorder, most recent episode (or current) mixed, severe, specified as with psychotic behavior
	ICD-10	F30.2	Manic episode, severe with psychotic symptoms
	ICD-10	F31.2	Bipolar disorder, current episode manic severe with psychotic features
	ICD-10	F31.5	Bipolar disorder, current episode depressed, severe, with psychotic features
	ICD-10	F31.64	Bipolar disorder, current episode mixed, severe, with psychotic features
	ICD-10	F32.3	Major depressive disorder, single episode, severe with psychotic features
	ICD-10	F33.3	Major depressive disorder, recurrent, severe with psychotic symptoms
Other psychotic disorders	ICD-9	290.8	Other specified senile psychotic conditions
	ICD-9	290.9	Unspecified senile psychotic condition
	ICD-9	293.81	Psychotic disorder with delusions in conditions classified elsewhere
	ICD-9	293.82	Psychotic disorder with hallucinations in conditions classified elsewhere
	ICD-10	F06.0	Psychotic disorder with hallucinations due to known physiological
	ICD-10	F06.2	Psychotic disorder with delusions due to known physiological condition

© 2023 Elser H et al. *JAMA Network Open.*

eTable 2. Typical and Atypical Antipsychotics

Typical Antipsychotics	Atypical Antipsychotics
Acetophenazine<br>Chlorpromazine<br>Chlorprothixene<br>Fluphenazine<br>Haloperidol<br>Loxapine<br>Mesoridazine<br>Molindone<br>Perphenazine<br>Pimozide<br>Prochlorperazine<br>Thiothixene<br>Trifluoperazine	Aripiprazole<br>Asenapine<br>Clozapine<br>Cariprazine<br>Iloperidone<br>Lurasidone<br>Olanzapine<br>Paliperidone<br>Risperidone<br>Quetiapine<br>Ziprasidone

© 2023 Elser H et al. *JAMA Network Open.*

eTable 3. Distribution of State Characteristics Overall and by Policy Type, 2003 to 2017

	**Overall**	**No policy**	**Medical, no retail outlets**	**Medical, retail outlets**	**Recreational, no retail outlets**	**Recreational, retail outlets**
State Months	9,180 (100.0)	6,144 (67.0)	1,469 (16.2)	1,259 (13.9)	125 (1.4)	138 (1.5)
States contributing time at risk	51 (100.0)	43 (84.0)	28 (55.0)	23 (45.0)	8 (16.0)	5 (10.0)
Percent Non-Hispanic Asian	3.6%	2.2%	7.8%	4.9%	5.9%	5.4%
Percent Non-Hispanic Black	11.0%	12.5%	8.6%	6.7%	15.9%	3.6%
Percent Hispanic	10.2%	8.3%	9.6%	18.5%	14.3%	15.6%
Percent Non-Hispanic White	71.5%	74.2%	66.4%	65.8%	57.7%	69.3%
Percent Unemployed	6.0%	5.8%	5.9%	7.1%	6.3%	5.4%
Percent Renters	33.7%	32.4%	35.8%	36.2%	44.0%	37.4%
Median Income	$50,048	$47,171	$55,500	$54,526	$66,023	$62,217

© 2023 Elser H et al. *JAMA Network Open.*

eTable 4. Crude Rates for Psychosis-Related Diagnoses and Prescriptions, 2003 to 2017

	**DIAGNOSES** [1]			**PRESCRIPTIONS** [2]		
	**Cases**	**Person-Months**	**Crude Rate** [3] (95% CI)	**Cases**	**Person-Months**	**Crude Rate** [3] (95% CI)
**No policy**	5,138,845	1,399,958,524	3.670 (3.667 – 3.674)	13,580,218	1,399,958,524	9.70 (9.69 – 9.71)
**Medical, no retail outlets**	863,828	172,068,754	5.020 (5.010, 5.031)	1,993,510	172,068,754	11.59 (11.57 – 11.60)
**Medical, retail outlets**	1,157,933	370,695,920	3.124 (3.118 – 3.129)	4,014,831	370,695,920	10.83 (10.82 – 10.84)
**Recreational, no retail outlets**	128,947	33,285,610	3.874 (3.853 – 3.895)	457,553	33,285,610	13.75 (13.71 – 13.79)
**Recreational, retail outlets**	214,354	39,180,898	5.471 (5.448 – 5.494)	753,172	39,180,898	19.22 (19.18 – 19.27)

1. Includes all unique diagnoses for schizophrenia, schizophreniform, and schizoaffective disorders; substance-related psychosis, mood disorder with psychotic features, and other forms of psychosis for each policy type over the study period.
2. Includes all unique prescriptions for first- and second-generation antipsychotics for each policy type over the study period.
3. Crude rates are calculated as the number of diagnoses or prescriptions, respectively, per 1,000 person-months of follow-up. 95% confidence intervals were calculated using Byar's approximation.

© 2023 Elser H et al. *JAMA Network Open.*

eTable 5. Adjusted Rate Ratios for the Association Between State Cannabis Policy Level and Psychosis-Related Diagnoses and Prescribed Antipsychotics, 2003 to 2017

	**Diagnoses** [1]	**Prescriptions** [2]
	**Rate Ratio** (95% CI) [3,4]	**Rate Ratio** (95% CI) [3,4]
**No policy**	(Ref)	(Ref)
**Medical, no retail outlets**	1.13 (0.94 – 1.36)	1.00 (0.88 – 1.13)
**Medical, retail outlets**	1.24 (0.96 – 1.61)	1.01 (0.87 – 1.19)
**Recreational, no retail outlets**	1.38 (0.93 – 2.04)	1.13 (0.84 – 1.51)
**Recreational, retail outlets**	1.39 (0.98 – 1.97)	1.14 (0.89 – 1.45)

1. Includes all unique diagnoses for schizophrenia, schizophreniform, and schizoaffective disorders; substance-related psychosis, mood disorder with psychotic features, and other psychosis by policy type.
2. Includes all unique prescriptions for first- and second-generation antipsychotics by policy type.
3. Rate ratios were calculated using negative binomial models with person-months at risk as the offset. Models were adjusted for state-level confounders including percent non-Hispanic Black, non-Hispanic Asian, and Hispanic; percent unemployed and percent renting their home; median income; and the overall claims rate. We included fixed effects for state, year, and calendar month to address spatial and temporal autocorrelation.
4. 95% CI were calculated with robust standard errors to account for repeated observations within states over time.

© 2023 Elser H et al. *JAMA Network Open.*

eTable 6. Adjusted Rate Ratios for the Association Between State Cannabis Policy Level and Psychosis-Related Diagnoses and Prescribed Antipsychotics by Sex, 2003 to 2017

	WOMEN		MEN	
	**Diagnoses**	**Prescriptions**	**Diagnoses**	**Prescriptions**
	RR (95% CI) [1,2]	RR (95% CI) [1,2]	RR (95% CI) [1,2]	RR (95% CI) [1,2]
**No policy**	(Ref)	(Ref)	(Ref)	(Ref)
**Medical, no retail outlets**	1.12 (0.93 – 1.34)	0.97 (0.86 – 1.10)	1.12 (0.93 – 1.35)	1.03 (0.90 – 1.17)
**Medical, retail outlets**	1.20 (0.92 – 1.56)	0.98 (0.85 – 1.14)	1.27 (0.89 – 1.65)	1.05 (0.88 – 1.25)
**Recreational, no retail outlets**	1.20 (0.79 – 1.84)	1.06 (0.80 – 1.42)	1.62 (1.08 – 2.41)	1.21 (0.90 – 1.64)
**Recreational, retail outlets**	1.36 (0.94 – 1.98)	1.08 (0.87 – 1.35)	1.42 (1.01 – 2.01)	1.21 (0.92 – 1.59)

1. Rate ratios were calculated using negative binomial models with person-months at risk as the offset. Models were adjusted for state-level confounders including percent non-Hispanic Black, non-Hispanic Asian, and Hispanic; percent unemployed and percent renting their home; median income; and the overall claims rate. We included fixed effects for state, year, and calendar month to address spatial and temporal autocorrelation.

2. 95% CI were calculated with robust standard errors to account for repeated observations within states over time.

© 2023 Elser H et al. *JAMA Network Open.*

eTable 7. Adjusted Rate Ratios for the Association Between State Cannabis Policy Level and Psychosis-Related Diagnoses and Prescribed Antipsychotics by Age Group, 2003 to 2017

	**Diagnoses** [1]	**Prescriptions** [2]
	**Rate Ratio** (95% CI) [3,4]	**Rate Ratio** (95% CI) [3,4]
**16 to 34 years**		
No policy	(Ref)	(Ref)
Medical, no retail outlets	1.02 (0.85 – 1.22)	0.98 (0.90 – 1.07)
Medical, retail outlets	1.13 (0.91 – 1.40)	1.00 (0.91 – 1.09)
Recreational, no retail outlets	1.36 (0.99 – 1.88)	1.02 (0.89 – 1.17)
Recreational, retail outlets	1.28 (0.73 – 2.26)	0.97 (0.74 – 1.28)
**35 to 54 years**		
No policy	(Ref)	(Ref)
Medical, no retail outlets	1.06 (0.82 – 1.36)	0.97 (0.82 – 1.15)
Medical, retail outlets	1.27 (0.92 – 1.74)	1.06 (0.87 – 1.30)
Recreational, no retail outlets	1.70 (1.05 – 2.77)	1.25 (0.95 – 1.65)
Recreational, retail outlets	1.55 (0.94 – 2.55)	1.16 (0.85 – 1.58)
**55 to 64 years**		
No policy	(Ref)	(Ref)
Medical, no retail outlets	1.13 (0.88 – 1.43)	0.99 (0.84 – 1.18)
Medical, retail outlets	1.47 (1.05 – 2.04)	1.11 (0.91 – 1.35)
Recreational, no retail outlets	2.03 (1.27 – 3.27)	1.38 (0.96 – 1.99)
Recreational, retail outlets	1.94 (1.21 – 3.12)	1.47 (1.13 – 1.92)
**65 years and older**		
No policy	(Ref)	(Ref)
Medical, no retail outlets	0.96 (0.82 – 1.13)	0.88 (0.73 – 1.05)
Medical, retail outlets	1.01 (0.81 – 1.27)	0.90 (0.72 – 1.13)
Recreational, no retail outlets	0.94 (0.63 – 1.39)	1.05 (0.74 – 1.49)
Recreational, retail outlets	1.24 (0.93 – 1.66)	1.33 (0.98 – 1.81)

1. Includes all unique diagnoses for schizophrenia, schizophreniform, and schizoaffective disorders; substance-related psychosis, mood disorder with psychotic features, and other psychosis by policy type.
2. Includes all unique prescriptions for first- and second-generation antipsychotics by policy type.
3. Rate ratios were calculated using negative binomial models with person-months at risk as the offset. Models were adjusted for state-level confounders including percent non-Hispanic Black, non-Hispanic Asian, and Hispanic; percent unemployed and percent renting their home; median income; and the overall claims rate. We included fixed effects for state, year, and calendar month to address spatial and temporal autocorrelation.
4. 95% CI were calculated with robust standard errors to account for repeated observations within states over time.

© 2023 Elser H et al. *JAMA Network Open.*

eTable 8. Adjusted Rate Ratios for the Association Between State Cannabis Policy Level and Psychosis-Related Diagnoses and Prescribed Antipsychotics by Age Group, 2003 to 2017

	WOMEN		MEN	
	**Diagnoses**	**Prescriptions**	**Diagnoses**	**Prescriptions**
	RR (95% CI) [1,2]	RR (95% CI) [1,2]	RR (95% CI) [1,2]	RR (95% CI) [1,2]
**16 to 34 years**				
No policy	(Ref)	(Ref)	(Ref)	(Ref)
Medical, no retail outlets	1.00 (0.85 – 1.17)	0.99 (0.92 – 1.07)	1.03 (0.84 – 1.27)	0.96 (0.88 – 1.05)
Medical, retail outlets	1.03 (0.84 – 1.27)	0.96 (0.88 – 1.06)	1.19 (0.96 – 1.49)	1.00 (0.90 – 1.10)
Recreational, no retail outlets	1.35 (0.88 – 2.08)	0.90 (0.80 – 1.01)	1.35 (0.96 – 1.91)	1.05 (0.87 – 1.26)
Recreational, retail outlets	1.36 (0.70 – 2.63)	0.88 (0.68 – 1.13)	1.19 (0.71 – 2.01)	0.94 (0.69 – 1.27)
**35 to 54 years**				
No policy	(Ref)	(Ref)	(Ref)	(Ref)
Medical, no retail outlets	1.04 (0.79 – 1.35)	0.95 (0.82 – 1.11)	1.04 (0.79 – 1.38)	0.99 (0.82 – 1.20)
Medical, retail outlets	1.19 (0.88 – 1.62)	1.01 (0.86 – 1.20)	1.30 (0.92 – 1.83)	1.04 (0.80 – 1.34)
Recreational, no retail outlets	1.44 (0.92 – 2.24)	1.11 (0.85 – 1.43)	1.91 (1.12 – 3.24)	1.24 (0.89 – 1.75)
Recreational, retail outlets	1.30 (0.76 – 2.25)	1.01 (0.77 – 1.33)	1.79 (1.11 – 2.88)	1.10 (0.76 – 1.59)
**55 to 64 years**				
No policy	(Ref)	(Ref)	(Ref)	(Ref)
Medical, no retail outlets	1.11 (0.88 – 1.41)	0.96 (0.82 – 1.11)	1.12 (0.87 – 1.44)	1.04 (0.87 – 1.24)
Medical, retail outlets	1.37 (1.01 – 1.85)	1.04 (0.86 – 1.25)	1.49 (1.04 – 2.14)	1.13 (0.92 – 1.39)
Recreational, no retail outlets	1.62 (0.99 – 2.64)	1.22 (0.86 – 1.72)	2.44 (1.47 – 4.06)	1.36 (0.94 – 1.97)
Recreational, retail outlets	1.66 (1.06 – 2.61)	1.27 (0.99 – 1.63)	2.32 (1.34 – 4.03)	1.42 (1.06 – 1.91)
**65 years and older**				
No policy	(Ref)	(Ref)	(Ref)	(Ref)
Medical, no retail outlets	0.97 (0.83 – 1.13)	0.86 (0.72 – 1.03)	0.93 (0.76 – 1.12)	0.92 (0.78 – 1.08)
Medical, retail outlets	1.02 (0.81 – 1.27)	0.88 (0.71 – 1.10)	1.02 (0.80 – 1.29)	0.96 (0.79 – 1.17)
Recreational, no retail outlets	0.90 (0.58 – 1.40)	0.99 (0.71 – 1.40)	1.09 (0.74 – 1.58)	1.09 (0.77 – 1.55)
Recreational, retail outlets	1.40 (1.03 – 1.89)	1.24 (0.92 – 1.69)	0.99 (0.72 – 1.37)	1.28 (0.97 – 1.70)

1. Rate ratios were calculated using negative binomial models with person-months at risk as the offset. Models were adjusted for state-level confounders including percent non-Hispanic Black, non-Hispanic Asian, and Hispanic; percent unemployed and percent renting their home; median income; and the overall claims rate. We included fixed effects for state, year, and calendar month to address spatial and temporal autocorrelation.
2. 95% CI were calculated with robust standard errors to account for repeated observations within states over time.

© 2023 Elser H et al. *JAMA Network Open.*

eTable 9. Adjusted Rate Ratios for the Association Between State Cannabis Policy Level and Psychosis-Related Diagnoses and Prescribed Antipsychotics by Race and Ethnicity, 2003 to 2017

	**Diagnoses** [1]	**Prescriptions** [2]
	**Rate Ratio** (95% CI) [3,4]	**Rate Ratio** (95% CI) [3,4]
**Asian** [5]		
No policy	(Ref)	(Ref)
Medical, no retail outlets	1.09 (0.92 – 1.29)	0.97 (0.83 – 1.14)
Medical, retail outlets	1.24 (0.98 – 1.57)	1.02 (0.86 – 1.22)
Recreational, no retail outlets	1.46 (1.08 – 2.38)	1.07 (0.76 – 1.49)
Recreational, retail outlets	1.61 (1.08 – 2.38)	1.22 (0.96 – 1.56)
**Black**		
No policy	(Ref)	(Ref)
Medical, no retail outlets	1.13 (0.85 – 1.52)	0.94 (0.76 – 1.17)
Medical, retail outlets	1.04 (0.77 – 1.39)	0.92 (0.74 – 1.15)
Recreational, no retail outlets	1.16 (0.70 – 1.92)	1.01 (0.69 – 1.49)
Recreational, retail outlets	1.43 (0.86 – 2.39)	0.97 (0.69 – 1.36)
**Hispanic**		
No policy	(Ref)	(Ref)
Medical, no retail outlets	1.03 (0.84 – 1.28)	1.01 (0.85 – 1.19)
Medical, retail outlets	1.28 (0.93 – 1.75)	1.09 (0.85 – 1.39)
Recreational, no retail outlets	1.38 (0.75 – 2.52)	1.08 (0.72 – 1.62)
Recreational, retail outlets	1.74 (1.09 – 2.78)	1.17 (0.87 – 1.57)
**White**		
No policy	(Ref)	(Ref)
Medical, no retail outlets	1.11 (0.94 – 1.32)	0.97 (0.86 – 1.08)
Medical, retail outlets	1.29 (1.00 – 1.66)	0.98 (0.86 – 1.12)
Recreational, no retail outlets	1.39 (0.97 – 1.99)	1.02 (0.81 – 1.29)
Recreational, retail outlets	1.45 (1.03 – 2.04)	1.11 (0.89 – 1.39)

1. Includes all unique diagnoses for schizophrenia, schizophreniform, and schizoaffective disorders; substance-related psychosis, mood disorder with psychotic features, and other psychosis by policy type.
2. Includes all unique prescriptions for first- and second-generation antipsychotics by policy type.
3. Rate ratios were calculated using negative binomial models with person-months at risk as the offset. Models were adjusted for state-level confounders including percent non-Hispanic Black, non-Hispanic Asian, and Hispanic; percent unemployed and percent renting their home; median income; and the overall claims rate. We included fixed effects for state, year, and calendar month to address spatial and temporal autocorrelation.
4. 95% CI were calculated with robust standard errors to account for repeated observations within states over time.
5. Because of sparsity of observations for Asian beneficiaries across covariate strata, we excluded observations from VT, SD, MT, and AK in order to calculate robust standard errors.

© 2023 Elser H et al. *JAMA Network Open.*

eTable 10. Adjusted Rate Ratios for Psychosis-Related Diagnoses by Subtype, 2003 to 2017

	**Non-affective psychotic disorders**	**Mood disorders with psychotic features**	**Substance-related psychosis**	**Other psychotic disorders**
	**Rate Ratio** (95% CI) [1,2]	**Rate Ratio** (95% CI) [1,2]	**Rate Ratio** (95% CI) [1,2]	**Rate Ratio** (95% CI) [1,2]
**No policy**	(Ref)	(Ref)	(Ref)	(Ref)
**Medical, no retail outlets**	1.13 (0.94 – 1.37)	1.06 (0.87 – 1.29)	1.09 (0.93 – 1.28)	1.16 (0.88 – 1.52)
**Medical, retail outlets**	1.24 (0.94 – 1.62)	1.06 (0.86 – 1.30)	1.14 (0.91 – 1.42)	1.62 (1.19 – 2.22)
**Recreational, no retail outlets**	1.37 (0.89 – 2.10)	1.08 (0.72 – 1.60)	1.29 (0.94 – 1.76)	1.07 (0.63 – 1.84)
**Recreational, retail outlets**	1.38 (0.97 – 1.97)	0.89 (0.54 – 1.47)	1.37 (0.93 – 2.03)	1.13 (0.31 – 4.08)

1. Rate ratios were calculated using negative binomial models with person-months at risk as the offset. Models were adjusted for state-level confounders including percent non-Hispanic Black, non-Hispanic Asian, and Hispanic; percent unemployed and percent renting their home; median income; and the overall claims rate. We included fixed effects for state, year, and calendar month to address spatial and temporal autocorrelation.
2. 95% CI were calculated with robust standard errors to account for repeated observations within states over time.

© 2023 Elser H et al. *JAMA Network Open.*

eTable 11. Adjusted Rate Ratios for the Association Between State Cannabis Policy Level for First-Generation and Second-Generation Antipsychotics, 2003 to 2017

	**First Generation** [1]	**Second Generation** [2]
	**Rate Ratio** (95% CI) [3,4]	**Rate Ratio** (95% CI) [3,4]
**No policy**	(Ref)	(Ref)
**Medical, no retail outlets**	1.07 (0.89 – 1.29)	0.99 (0.87 – 1.12)
**Medical, retail outlets**	1.04 (0.82 – 1.32)	1.01 (0.87 – 1.17)
**Recreational, no retail outlets**	1.14 (0.77 – 1.70)	1.12 (0.84 – 1.48)
**Recreational, retail outlets**	1.14 (0.77 – 1.70)	1.12 (0.89 – 1.43)

1. First-generation antipsychotics included acetophenazine, chlorpromazine, chlorprothixene, fluphenazine, haloperidol, loxapine, mesoridazine, molindone, perphenazine, pimozide, prochlorperazine, thiothixene, and trifluoperazine.
2. Second-generation antipsychotics included aripiprazole, asenapine, cariprazine, clozapine, iloperidone, lurasidone, olanzapine, paliperidone, quetiapine, risperidone, and ziprasidone.
3. Rate ratios were calculated using negative binomial models with person-months at risk as the offset. Models were adjusted for state-level confounders including percent non-Hispanic Black, non-Hispanic Asian, and Hispanic; percent unemployed and percent renting their home; median income; and the overall claims rate. We included fixed effects for state, year, and calendar month to address spatial and temporal autocorrelation.
4. 95% CI were calculated with robust standard errors to account for repeated observations within states over time.

© 2023 Elser H et al. *JAMA Network Open.*

eTable 12. Sensitivity Analyses With Total Diagnoses and Prescriptions as Offsets

	**Diagnoses** [1]	**Prescriptions** [2]
	**Rate Ratio** (95% CI) [3,4]	**Rate Ratio** (95% CI) [3,4]
**No policy**	(Ref)	(Ref)
**Medical, no retail outlets**	1.11 (0.97 – 1.28)	0.98 (0.89 – 1.07)
**Medical, retail outlets**	1.21 (0.97 – 1.50)	0.95 (0.85 – 1.06)
**Recreational, no retail outlets**	1.39 (1.00 – 1.95)	0.98 (0.79 – 1.22)
**Recreational, retail outlets**	1.64 (1.16 – 2.31)	0.92 (0.79 – 1.07)

1. Diagnoses for all medical conditions for each state-month were summed and specified as the offset.
2. All filled prescriptions for each state-moth were summed and specified as the offset.
3. Rate ratios were calculated using negative binomial models. Models were adjusted for state-level confounders including percent non-Hispanic Black, non-Hispanic Asian, and Hispanic; percent unemployed and percent renting their home; median income; and the overall claims rate. We included fixed effects for state, year, and calendar month to address spatial and temporal autocorrelation.
5. 95% CI were calculated with robust standard errors to account for repeated observations within states over time.

© 2023 Elser H et al. *JAMA Network Open.*

eTable 13. Sensitivity Analysis With Simplified 3-Category Exposure

	Diagnoses	Prescriptions
	**Rate Ratio** (95% CI) [1,2]	**Rate Ratio** (95% CI) [1,2]
**No policy**	(Ref)	(Ref)
**Medical**	1.17 (0.96 – 1.42)	1.00 (0.89 – 1.14)
**Recreational**	1.33 (0.98 – 1.82)	1.13 (0.89 – 1.42)

1. Rate ratios were calculated using negative binomial models with person-months at risk as the offset. Models were adjusted for state-level confounders including percent non-Hispanic Black, non-Hispanic Asian, and Hispanic; percent unemployed and percent renting their home; median income; and the overall claims rate. We included fixed effects for state, year, and calendar month to address spatial and temporal autocorrelation.
2. 95% CI were calculated with robust standard errors to account for repeated observations within states over time.

© 2023 Elser H et al. *JAMA Network Open.*

eTable 14. Sensitivity Analysis With Recreational Policies With 6-Category Exposure

	**Diagnoses**	**Prescriptions**
	**Rate Ratio** (95% CI) [1,2]	**Rate Ratio** (95% CI) [1,2]
**No policy**	(Ref)	(Ref)
**Medical, no retail outlets**	1.13 (0.94 – 1.35)	1.00 (0.88 – 1.13)
**Medical, retail outlets**	1.25 (0.96 – 1.62)	1.02 (0.87 – 1.19)
**Recreational, no retail outlets**	1.39 (0.94 – 2.06)	1.13 (0.85 – 1.51)
**Recreational, retail outlets, restrictions**	1.61 (1.17 – 2.21)	1.19 (0.91 – 1.55)
**Recreational, retail outlets, no restrictions**	0.91 (0.65 – 1.26)	1.02 (0.78 – 1.34)

1. Six-category version of the exposure variable separates states with recreational policies that allow retail outlets into those with and without THC dose-related restrictions defined as THC dose per serving size, THC content per package, or product types (e.g., bans on edible products)
2. Rate ratios were calculated using negative binomial models with person-months at risk as the offset. Models were adjusted for state-level confounders including percent non-Hispanic Black, non-Hispanic Asian, and Hispanic; percent unemployed and percent renting their home; median income; and the overall claims rate. We included fixed effects for state, year, and calendar month to address spatial and temporal autocorrelation.
3. 95% CI were calculated with robust standard errors to account for repeated observations within states over time.

© 2023 Elser H et al. *JAMA Network Open.*

eTable 15. Sensitivity Analysis Restricted to State-Months With Policy in Place

	**Diagnoses**	**Prescriptions**
	**Rate Ratio** (95% CI) [1,2]	**Rate Ratio** (95% CI) [1,2]
**Medical, no retail outlets**	(Ref)	(Ref)
**Medical, retail outlets**	1.11 (0.94 – 1.31)	1.06 (0.95 – 1.19)
**Recreational, no retail outlets**	1.41 (1.00 – 1.99)	1.28 (1.04 – 1.59)
**Recreational, retail outlets**	1.29 (0.96 – 1.72)	1.19 (0.98 – 1.45)

1. Rate ratios were calculated using negative binomial models with person-months at risk as the offset. Models were adjusted for state-level confounders including percent non-Hispanic Black, non-Hispanic Asian, and Hispanic; percent unemployed and percent renting their home; median income; and the overall claims rate. We included fixed effects for state, year, and calendar month to address spatial and temporal autocorrelation.
2. 95% CI were calculated with robust standard errors to account for repeated observations within states over time.

eTable 16. Negative Outcome Controls—All Diagnoses and All Prescriptions

	**Diagnoses** [1]	**Prescriptions** [2]
	**Rate Ratio** (95% CI) [3,4]	**Rate Ratio** (95% CI) [3,4]
**No policy**	(Ref)	(Ref)
**Medical, no retail outlets**	1.01 (0.94 – 1.08)	1.01 (0.93 – 1.11)
**Medical, retail outlets**	0.96 (0.87 – 1.06)	1.04 (0.92 – 1.18)
**Recreational, no retail outlets**	0.89 (0.77 – 1.03)	1.13 (0.95 – 1.34)
**Recreational, retail outlets**	0.75 (0.65 – 0.87)	1.26 (1.00 – 1.60)

1. Diagnoses for all medical condition were specified as the outcome of interest.
2. All filled prescriptions were specified as the outcome of interest.
3. Rate ratios were calculated using negative binomial models with person-months at risk as the offset. Models were adjusted for state-level confounders including percent non-Hispanic Black, non-Hispanic Asian, and Hispanic; percent unemployed and percent renting their home; median income; and the overall claims rate. We included fixed effects for state, year, and calendar month to address spatial and temporal autocorrelation.
4. 95% CI were calculated with robust standard errors to account for repeated observations within states over time.

© 2023 Elser H et al. *JAMA Network Open.*

eTable 17. Negative Exposure Control—Hypothetical Law Change

	**Diagnoses**	**Prescriptions**
	**Rate Ratio** (95% CI) [3,4]	**Rate Ratio** (95% CI) [3,4]
**No policy**	(Ref)	(Ref)
**Medical, no retail outlets**	1.00 (0.98 – 1.02)	1.00 (0.98 – 1.01)
**Medical, retail outlets**	1.00 (0.98 – 1.02)	1.00 (0.98 – 1.01)
**Recreational, no retail outlets**	1.00 (0.95 – 1.05)	1.01 (0.97 – 1.04)
**Recreational, retail outlets**	0.97 (0.92 – 1.03)	1.00 (0.96 – 1.03)

1. State cannabis policy levels were randomly re-assigned at the state-month to create a permutation of observed policy changes that served as the negative exposure control for this analysis.
2. Rate ratios were calculated using negative binomial models with person-months at risk as the offset. Models were adjusted for state-level confounders including percent non-Hispanic Black, non-Hispanic Asian, and Hispanic; percent unemployed and percent renting their home; median income; and the overall claims rate. We included fixed effects for state, year, and calendar month to address spatial and temporal autocorrelation.
3. 95% CI were calculated with robust standard errors to account for repeated observations within states over time.

© 2023 Elser H et al. *JAMA Network Open.*

eTable 18. Negative Exposure Control—Naloxone Overdose Prevention Laws

	Diagnoses	Prescriptions
	**Rate Ratio** (95% CI) [1,2]	**Rate Ratio** (95% CI) [1,2]
**No law**	(Ref)	(Ref)
**Overdose prevention law**	1.13 (1.02 – 1.25)	1.15 (1.06 – 1.25)

1. Rate ratios were calculated using negative binomial models with person-months at risk as the offset. Models were adjusted for state-level confounders including percent non-Hispanic Black, non-Hispanic Asian, and Hispanic; percent unemployed and percent renting their home; median income; and the overall claims rate. We included fixed effects for state, year, and calendar month to address spatial and temporal autocorrelation.

2. 95% CI were calculated with robust standard errors to account for repeated observations within states over time.

eTable 19. Estimated Average Effect of Treatment on Treated (ATT) Comparing Sequential Levels of State Cannabis Policies Using Methods Proposed by Callaway and Sant'Anna, 2003 to 2017 [1]

	**DIAGNOSES** [2] ATT (95% CI)	**PRESCRIPTIONS** [3] ATT (95% CI)
**Medical, no retail outlets vs. no policy**	- 3.64 (-16.80, 9.53)	- 7.24 (-23.51, 9.03)
**Medical, retail outlets vs. Medical, no retail outlets**	41.47 (-39.20, 122.15	69.65 (-57.85, 197.16)
**Recreational, no retail outlets vs. Medical, retail outlets**	- 0.44 (-14.58, 13.70)	16.18 (-10.22, 42.58)
**Recreational, retail outlets vs. Recreational, no retail outlets**	5.00 (-8.15, 18.14)	4.13 (-12.75, 21.01)

1. Because the estimator is built for binary treatments, we estimate a series of contrasts for each sequential policy level, restricting each analysis to the state-months with only those two policy levels, and allowing for unbalanced panel data.
2. Includes all unique diagnoses for schizophrenia, schizophreniform, and schizoaffective disorders; substance-related psychosis, mood disorder with psychotic features, and other forms of psychosis for each policy type over the study period.
3. Includes all unique prescriptions for first- and second-generation antipsychotics for each policy type over the study period.

© 2023 Elser H et al. *JAMA Network Open.*
