# Supplementary material for: State Cannabis Legalization and Psychosis-Related Health Care Utilization
Source: JAMA Netw Open. 2023 Jan 25;6(1):e2252689. doi: 10.1001/jamanetworkopen.2022.52689 (PMC9925044; doi:10.1001/jamanetworkopen.2022.52689)
Supplement: Supplement 2. — Data Sharing Statement [file jamanetwopen-e2252689-s002.pdf]

Data Sharing Statement

Elser. State Cannabis Legalization and Psychosis-Related Health Care Utilization. *JAMA Netw Open*. Published January 25, 2023. doi:10.1001/jamanetworkopen.2022.52689

Data
Data available: No
